# A physically and mentally active lifestyle relates to younger brain and cognitive age

**DOI:** 10.1007/s11357-025-01764-w

**Published:** 2025-07-07

**Authors:** Niklas Behrenbruch, Svenja Schwarck, Beate Schumann-Werner, Eóin N. Molloy, Berta Garcia-Garcia, Anne Hochkeppler, Larissa Fischer, Anna-Therese Büchel, Enise I. Incesoy, Jose Bernal, Niklas Vockert, Patrick Müller, Gusalija Behnisch, Bárbara Morgado, Hermann Esselmann, Constanze I. Seidenbecher, Björn H. Schott, Henryk Barthel, Osama Sabri, Jens Wiltfang, Michael C. Kreissl, Emrah Düzel, Anne Maass

**Affiliations:** 1 German Center for Neurodegenerative Diseases (DZNE), Leipziger Str. 44, Haus 64, 39120 Magdeburg, Germany; 2 Faculty of Natural Sciences, Otto Von Guericke University Magdeburg, 39106 Magdeburg, Germany; 3 Institute of Cognitive Neurology and Dementia Research (IKND), Otto Von Guericke University Magdeburg, 39120 Magdeburg, Germany; 4 Division of Nuclear Medicine, Department of Radiology & Nuclear Medicine, Faculty of Medicine, Otto Von Guericke University Magdeburg, 39120 Magdeburg, Germany; 5 Department of Neurobiology and Behavior, University of California, Irvine, Irvine, 92697 USA; 6 Centre for Clinical Brain Sciences, The University of Edinburgh, Edinburgh, EH16 4SB UK; 7 Division of Cardiology and Angiology, University Hospital Magdeburg, 39120 Magdeburg, Germany; 8 Leibniz Institute for Neurobiology (LIN), 39118 Magdeburg, Germany; 9 Department for Psychiatry and Psychotherapy, University Medical Center Göttingen (UMG), 37075 Göttingen, Germany; 10 Center for Behavioral Brain Sciences (CBBS), Magdeburg, Germany; 11 German Center for Neurodegenerative Diseases (DZNE), 37075 Göttingen, Germany; 12 Department of Nuclear Medicine, University Hospital Leipzig, 04103 Leipzig, Germany; 13 Neurosciences & Signaling Group, Institute of Biomedicine (iBiMED), Department of Medical Sciences, University of Aveiro, 3810-193 Aveiro, Portugal

**Keywords:** Brain age, Cognitive age, Resilience, Brain maintenance, Alzheimer’s disease, Lifestyle

## Abstract

Resistance to age-related pathological changes (brain maintenance), including Alzheimer’s disease, cerebrovascular disease, and neurodegeneration may promote cognitive resilience in aging. However, how lifestyle and health profiles relate to successful cognitive and brain aging remains poorly understood. In a novel, deeply phenotyped cohort of 211 cognitively unimpaired older adults (age = 71.0 ± 7.4 years, 46% female), we characterized principal components of lifestyle and health using questionnaire, fitness, and blood data. We estimated cognitive age gap (CAG) based on comprehensive neuropsychological data and brain age gap (BAG) based on brain-pathology markers, including plasma biomarkers of Alzheimer’s pathology (pTau_217_ and Aβ_1-42_/Aβ_1-40_), MRI-based measures of white matter hyperintensities, perivascular spaces, and brain atrophy. Regression analyses tested how the observed lifestyle-health profiles were related to CAG and BAG. Seven principal components explained 49% of the variance in health and lifestyle. The second component, characterized by a mentally and physically active life and low cardiovascular risk, was associated with lower CAG (*β* = − 0.66, *p* < 0.001) and BAG (*β* = − 0.52, *p* = 0.003), reflecting a younger-than-expected brain and cognitive age, respectively. The association of an active lifestyle and lower CAG was partially mediated by BAG. Higher CAG was also associated with other lifestyle components characterized by low mental stimulation. APOE-ε4 carriers exhibited higher BAG. In conclusion, a lifestyle combining low cardiovascular risk, high mental engagement throughout life and high physical activity/fitness is jointly associated with less-than-expected brain pathology and better-than-expected cognitive performance, supporting its involvement in brain maintenance and cognitive resilience to aging.

**Supplementary Information:**

The online version contains supplementary material available at 10.1007/s11357-025-01764-w.

## Introduction

While some cognitive decline in old age is “normal” (seen on average), there is also large inter-individual variability in aging and some older individuals perform much better than expected for their age [1, 2]. Cognitive resilience against age-related cognitive decline may be explained by mechanisms, such as brain maintenance and cognitive reserve [3–6] (see also https://reserveandresilience.com/framework/, accessed 04/10/2025). Brain maintenance refers to resistance against age-associated pathological brain changes, including the accumulation of Alzheimer’s-associated proteins, white matter lesions, enlarged perivascular spaces, and gray matter atrophy [7–10]. On the other hand, cognitive reserve may allow some individuals to cope with pathology and maintain cognition despite aging [11, 12]. Determining the factors that promote successful cognitive and brain aging, especially modifiable ones, is crucial for prevention and intervention.

Various lifestyle and health factors, including education, intellectually stimulating activities, physical activity, and avoidance of alcohol and smoking, have been individually linked to successful cognitive aging; however, results have been mixed [13, 14] (for reviews, see, e.g., [1, 15]). Most studies have used self-reported lifestyle or health questionnaires, while only a few used objective measures (e.g., muscle strength [16] or blood sugar levels [17, 18]). Furthermore, lifestyle and health profiles in old age are multifaceted and different combinations of early life experience (e.g., low education or mental stimulation), mental and physical activity, and current health status across physical and psychological domains exist. These profiles may be differentially associated with cognitive resilience mechanisms. Given the multifaceted nature of these factors [16, 19], multivariate approaches such as principal component analysis (PCA) can help to identify latent structures by capturing shared variance. However, how specific health/lifestyle profiles or phenotypes in cognitively unimpaired elderly are linked to cognitive resilience and, in particular, brain maintenance is not well understood.

In the current cross-sectional observational study, we aimed to determine health and lifestyle profiles in a newly established aging cohort and how these are associated with proxies of brain maintenance and cognitive resilience to aging more generally. While age-related cognitive resilience mechanisms are optimally studied longitudinally, these can be approximated by determining cross-sectional deviations from the expected pathological brain change and cognitive performance for a given age. If there is sufficient variability in age among the participants, methods that extract latent components from brain and cognitive outcomes, with the goal of maximizing covariance with age, may provide insight into age-related changes. In this context, previous studies applied computational models and machine learning to predict brain age based on gray matter volume/thickness [18, 20, 21] and partial least squares regression to predict cognitive age based on cognitive test scores [22–24]. The difference between predicted brain or cognitive age and chronological age has been referred to as brain age gap (BAG) or cognitive age gap (CAG), respectively. Evidence for construct validity of these deviations comes from studies demonstrating that higher BAG and CAG is associated with accelerated biological aging, subsequent cognitive decline over time [23, 25] and the conversion to cognitive impairment [26, 27].

While CAG may be a proxy of overall age-related cognitive resilience, BAG may rather reflect brain maintenance factors. Notably, previous models of BAG were based on gray or white matter volumes, diffusion MRI [28], and, more recently, functional MRI data [29]. However, these models on BAG lacked the inclusion of common age-related brain pathology measures such as tau and amyloid-beta accumulation, white matter hyperintensities (WMH), and perivascular spaces (PVS). Apart from lifestyle, genetics may also explain differences in cognitive and brain aging. One potential candidate is the apolipoprotein E (*APOE*) genotype, namely the ε4 allele, which is a risk factor for sporadic Alzheimer’s disease (AD) [30, 31] and overall brain aging [32]. Thus, we also examined whether the carriage of the *APOE* ε4 allele is related to older cognitive or brain age in our cognitively unimpaired older adults.

The main goals of the current study were (i) to determine cognitive age and brain age based on comprehensive cognitive data and various brain-pathology proxies, respectively; (ii) to characterize our new aging cohort in terms of lifestyle and health profiles; and (iii) to examine whether lifestyle/health profiles and *APOE* ε4 genotype are associated with cognitive and brain age gap. Therefore, we applied multivariate analysis approaches. To estimate brain age, we established a novel approach by not only including conventionally used measures for brain age estimation such as cortical thickness but also measures of age-related pathology such as plasma biomarkers of Alzheimer’s pathology (pTau_217_ and Aβ_1–42_/Aβ_1–40_ ratio) and MR-based proxies of white matter lesions and PVS in basal ganglia and centrum semiovale into our model.

## Methods

### Study overview and cohort

**Fig. 1:**
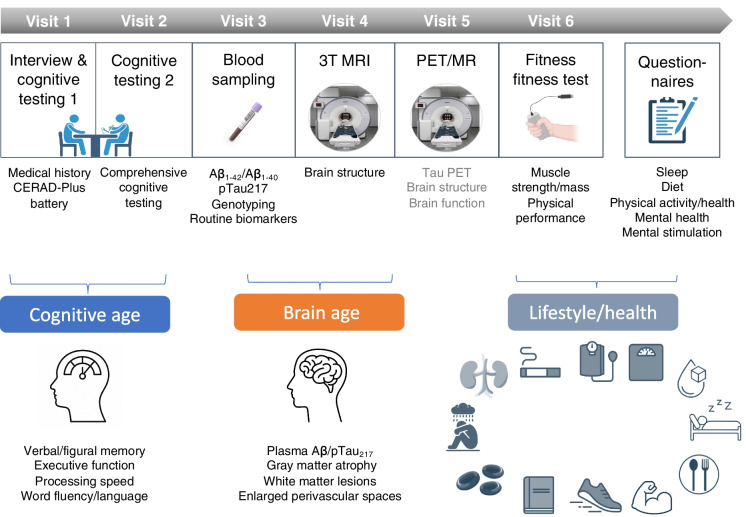
Study overview and key measures. The study (central project Z03 of the CRC1436) included up to 6 visits. All participants performed two comprehensive cognitive testing sessions (visit 1 and 2) and blood sampling (visit 3). Most participants (ca. 80%) underwent MR scanning at 3 Tesla including a T1- and T2-weighted sequence among others (visit 4 and 5). A subsample (ca. 42%) also performed Tau PET imaging at a 3 T PET/MR scanner using [^18^F]PI-2620, while additional structural and functional MR sequences were acquired (visit 5). A physical fitness test was performed to assess muscular (and aerobic) fitness (visit 6). Finally, participants filled out various questionnaires related to health and lifestyle or life experience

The current study presents the first baseline data of a novel ongoing aging cohort that is build up in the central project “Z03—Human molecular imaging aging and SuperAging cohort” as part of the Collaborative Research Centre (CRC) 1436 “Neural Resources of Cognition” (www.sfb.1436.de). Briefly, the central project aims to establish a deeply phenotyped cohort of healthy older adults to study the factors and mechanisms underlying successful brain and cognitive aging. Participants are characterized based on cognition, brain structure and function, lifestyle factors, health, fitness, molecular markers, and genetic factors as shown in Fig. 1. A subsample of participants also underwent PET/MR using [^18^F]PI-2620 to assess regional tau accumulation. However, the PET scan was not obligatory for study inclusion. As part of the Z03 project, the deeply phenotyped participants are further allocated to specific subprojects within the CRC1436, with a focus on training-related plasticity; however, the present study exclusively examined data from the Z03 assessments. The study design and protocol were approved by the local Ethics Committee of the Medical Faculty, Otto-von-Guericke University Magdeburg (200/19) and were carried out in accordance with the code of ethics of the World Medical Association (Declaration of Helsinki, 1967). Written informed consent was obtained from all participants. Study registration to the “German Clinical Trials Register” https://drks.de has been submitted and the registration is currently processed (Clinical trial number: not applicable). We note that our study employed a strictly observational design and did not involve any interventions or treatment.

Cognitively unimpaired community-dwelling older adults aged 60 years or older were recruited in and around the city of Magdeburg. General inclusion criteria were no objective cognitive decline (according to age-, sex- and education-adjusted norms of the CERAD-Plus Neuropsychological Assessment Battery; see below), no neurological or psychiatric conditions, lack of major medical illness, no diagnosis of diabetes, no intake of medications that might affect cognition (e.g., antidepressant medication, sleep or certain pain medication), and no substance-related addiction. For the MRI and PET measurements, further eligibility criteria were applied (see Supplementary Methods - MRI and PET scanning). Participants were recruited via a media campaign, newspaper advertisements, word-of-mouth as well as with a supportive letter campaign of the city of Magdeburg. Between 2021 and 2024, in total 18,000 randomly selected elderly individuals were contacted via letter and invited to sign up for the study. Response rate was 5.8%. An initial screening was performed via telephone and potentially eligible participants were invited for the first visit that included an interview/anamnesis and neuropsychological testing. An overview of the sample selection is given in Supplementary Figure S1. Briefly, 348 individuals were enrolled in the study (as of 12/12/2024) who had performed both cognitive testing sessions at that time. Of these, 275 had completed at least the first 3 T MRI scan (visit 4), 268 had plasma AD biomarker information available, and 211 had completed a physical fitness assessment including measures of muscular health. A subsample of 148 participants had performed the Tau PET scan (ca. 42%), which was not analyzed here. Final analyses were performed on 206 participants with estimates of CAG and lifestyle/health profiles and 171 with estimates of BAG and lifestyle/health profiles, as described below.

### Visit 1 and 2: interview and cognitive testing

After enrollment, the following personal and medical information was collected during visit 1: demographics (age, sex, education, occupational background), marital status and living situation, medical history (cardiovascular risk factors, comorbidities, current medications), hearing and vision impairment, and handedness. Cognitive testing was conducted on two different days. During visit 1, participants underwent the CERAD-Plus (Consortium to Establish a Registry for Alzheimer’s Disease) Neuropsychological Assessment Battery to ensure unimpaired cognitive status. The CERAD-Plus includes the following measures: Verbal Fluency (semantic fluency, animals); Confrontational Naming (15-item short form of the Boston Naming Test); Mini-Mental State Examination (MMSE); Word List Memory/Learning (10 nouns administered on three successive occasions, each time in a different order); Constructional Praxis/Figures (copying four designs); Word List Delayed Recall (free recall of the 10 nouns presented earlier); Word List Recognition (recognition of the 10 nouns presented earlier, embedded with 10 foils); Recall of Constructional Praxis/Figures (free recall of the four designs presented earlier); Trail Making Test (TMT) A and B; Verbal Fluency (phonetic fluency, words starting with “S”).

To assess unimpaired cognitive status and determine inclusion in the study, raw scores of each subtest were transformed into age-, sex-, and education-adjusted z-scores. In previous studies, a z-score < − 1.5 was recommended as a cutoff for low cognitive performance associated with mild cognitive impairment (MCI) [33]. However, it has been shown that there is a high percentage of one or more low test scores in the CERAD Neuropsychological Assessment Battery, possibly leading to misdiagnosis of cognitive impairment [34]. Therefore, in addition to the exclusion criterion of a MMSE score below 26, the following more comprehensive exclusion criteria were defined by the multidisciplinary study team: a z-score smaller than − 1.5 SD in at least one of the tests from the CERAD-Plus test battery: Verbal Fluency (animals); Boston Naming Test; Word List Total; Word List Delayed Recall; Word List Recognition; Figures - Copy; Figures - Saving; Verbal Fluency (S-words), TMT-A; TMT-B and evidence of possible cognitive limitations based on the neuropsychological Jak/Bondi criteria [35]. An exclusion due to Jak/Bondi criteria was warranted if participants exhibited either (i) at least two test performances below − 1 SD within the same cognitive domain or (ii) at least one test performance below − 1 SD in at least one test across each of the three cognitive domains (see Supplementary Methods for domain mapping).

Participants who met these cognitive exclusion criteria were, if necessary, referred to a memory clinic for further neuropsychological evaluation.

During visit 2, participants received a further comprehensive cognitive testing of different cognitive domains applying the following tests: LEARNING AND VERBAL MEMORY: (1) Verbal Learning and Memory Test (VLMT) [36], a German version of Rey’s Auditory Verbal Learning Test including five repetitions of free recall, an interference list followed by an immediate recall as well as a delayed recall after 20–30 min followed by a recognition task; (2) Logical Memory (LM) I & II of the Wechsler Memory Scale (WMS) [37] including immediate recall, delayed recall and a recognition task; (3) German picture version of the Free and Cued Selective Reminding Test (FCSRT) [38], which provides controlled learning during the study phase and results into a sum score of free and cued immediate recall (total immediate recall); VISUOSPATIAL CONSTRUCTION AND MEMORY: (4) Rey Complex Figure Test and Recognition Trial (RCFT) [39] assessing visuospatial constructional ability, immediate and delayed recall, recognition memory, as well as processing speed; ATTENTION: (5) Symbol Digit Modalities Test (SDMT) [40] as a measure of information processing speed; (6) Go/No-go task of the Test battery for Attentional Performance [41] as a measure of response inhibition; EXECUTIVE FUNCTION AND LANGUAGE: (7) three semantic and two phonematic verbal fluency tasks including a switching task in each domain, based on the German Regensburger Wortflüssigkeitstest (RWT) [42], which is equivalent to the English Controlled Oral Word Association Test (COWAT).

The time interval between the two cognitive assessments was approximately 7 days [median: 7 days, (Q1, Q3): (7, 11) days].

### Visit 3: blood sampling and routine blood diagnostics

Fasting blood samples were taken between 8 and 9 am at visit 3. A total of 12 venous blood samples were taken by trained study assistants (6 × ethylenediaminetetraacetic acid (EDTA), 2 × serum, 2 × lithium-heparin, 1 × PaxGene RNA, 1 × citrate), amounting a total blood volume of ~ 100 ml. Two EDTA samples and one lithium-heparin sample were cooled, and delivered to a local clinical routine laboratory (Institute for Clinical Chemistry and Pathobiochemistry, Medical Faculty, Otto-von-Guericke-University Magdeburg) within 30 min after blood drawing. Several standard blood laboratory parameters were measured, including homocysteine, estimated glomerular filtration rate (eGFR), albumin, leukocyte count, hemoglobin, platelet count, and hemoglobin A1c (HbA1c). Two EDTA samples were centrifuged with 2000 × *g* at room temperature (RT) for 10 min after which the buffy coat was extracted and stored at 4 °C (to be sent out for genotyping later). The blood plasma was aliquoted and frozen at – 80 °C (to be sent out for AD biomarkers assessment later). Likewise, all other blood materials were stored at − 80 °C after processing and aliquoting.

The median time interval between the blood sampling and cognitive testing 2 was 25 days [(Q1, Q3): (14, 97) days].

#### Assessment of Alzheimer’s disease plasma biomarkers

Blood biomarkers for amyloid and tau pathology were analyzed at DZNE/University Göttingen. Aliquots of 500 μl EDTA–blood plasma stored at − 80 °C in Matrix 0.5-mL tubes (Thermo Scientific) were thawed at RT, mixed vigorously for 5–10 s and centrifuged for 10 min at 10,000 × *g* at RT in a fixed angle rotor. The concentrations of Aβ_1–40_, Aβ_1–42_ and pTau_217_ were determined using the commercially available plasma Aβ_1–40_, Aβ_1–42_, and pTau_217_ Immunoreaction Cartridges on the fully automated Lumipulse G600II System as previously described [43]. Manufacturer quality control material was analyzed before and after testing each day and determined to be within the manufacturer’s specifications. Precision based on quality control data was < 5% CV for all Lumipulse assays tested. Internal quality control samples were included in all runs to assess inter-assay variability. EDTA-plasma was introduced into the instrument using individual 2 ml screw cap micro-tubes (Sarstedt, Germany). All assays were performed as single measurements following the kit instructions.

#### APOE genotyping

Genotyping analyses were performed at the Leibniz Institute for Neurobiology (Magdeburg). *APOE* genotyping was carried out using a polymerase chain reaction (PCR)-based approach followed by restriction fragment length polymorphism (RFLP) analysis. Genomic DNA was extracted from whole blood using the QIAamp® DNA Blood Mini Kit and amplified with primers targeting a 318 base pair region of the *APOE* gene. The PCR reaction mixture contained Taq polymerase, Taq buffer, deoxynucleoside triphosphates (dNTPs), and specific primers. The amplification protocol involved an initial denaturation at 94 °C for 3 min, followed by 40 cycles of denaturation at 94 °C for 10 s, annealing at 65 °C for 30 s, and extension at 72 °C for 30 s, with a final extension at 72 °C for 7 min. The resulting amplicons were digested with AflIII or HaeII restriction enzymes, and the digestion patterns were analyzed by electrophoresis on a 3% agarose gel. Genotypes were identified based on the distinct fragment patterns associated with each *APOE* allele: *APOE* ε2/2 (231 bp, 267 bp), *APOE* ε2/3 (231 bp, 231 and 267 bp), *APOE* ε2/4 (231 and 295 bp, 231 and 267 bp), *APOE* ε3/3 (231 bp, 231 bp), *APOE* ε3/4 (231 and 295 bp, 231 bp), and *APOE* ε4/4 (295 bp, 231 bp). Participants were classified as *APOE* ε4 non-carriers (those lacking the ε4 allele) or carriers (those with at least one ε4 allele). Additional genotyping was performed for Klotho (rs9536314) and KIBRA (rs17070145), which were not analyzed in this study.

### Visit 4 and 5: MRI acquisition and processing

#### Acquisition

Participants underwent a 3 T MRI session (visit 4) using a Siemens SKYRA scanner and a combined PET/MR session with a Siemens MAGNETOM Biograph scanner (visit 5). The SKYRA session covered standard sequences (T1, Fluid-Attenuated Inversion Recovery (FLAIR), Fast Low Angle Shot) as well as Diffusion Tensor Imaging and Quantitative Susceptibility Mapping. The MR sequences of the second session (visit 5) focused on more specific brain targets, including resting-state fMRI, perfusion, angiography, and structural integrity of the medial temporal lobe and locus coeruleus, and their analysis was not within the scope of this study. While all subjects who were willing and suitable for MRI were scheduled for both MRI sessions, the additional Tau PET acquisition as part of the PET/MR session was explicitly left to the subjects’ discretion and was only performed after separate verification of PET eligibility. Only 148 of all participants underwent Tau PET and PET measures were not included in the current analyses (i.e., for estimation of BAG).

In the current study, we included volumetric measures based on the multi-echo MPRAGE (MEMPRAGE) and FLAIR sequence from the SKYRA 3 T MRI. The 0.8 mm isotropic MEMPRAGE sequence was acquired with the following parameters: repetition time (TR) = 2.56 s, echo times (TE_1_ = 1.85 ms, TE_2_ = 3.75 ms, TE_3_ = 5.65 ms, TE_4_ = 7.55 ms), inversion time (TI) = 1.1 s, and flip angle = 7°, and a total acquisition time of 6 min 52 s. The four echoes were combined into a single image using a magnitude-based combination approach to optimize signal-to-noise ratio and tissue contrast. The FLAIR sequence was acquired with the following parameters: 1.0 mm isotropic, TR = 5000 ms, TE = 393 ms, TI = 1800 ms, and a total acquisition time of 4 min 37 s. The median interval between MRI and the second cognitive testing session was 48 days [(Q1, Q3): (26, 93) days].

#### Processing

##### FreeSurfer Segmentation for thickness and volume measures

T1-weighted MPRAGE scans were segmented using FreeSurfer version 7.1; https://surfer.nmr.mgh.harvard.edu/, accessed 04/10/2025) using the Desikan–Killiany atlas. The average gray matter thickness across the entire brain, along with the bilateral hippocampal volumes and cerebrospinal fluid (CSF) volumes, were used to estimate brain age (see below). All volumes were adjusted for total intracranial volumes (TICV). TICV were estimated using FreeSurfer’s SAMSEG-based structural segmentation, rather than conventional atlas-based estimates, which are biased towards total brain volume[44].

##### Estimation of perivascular spaces and white matter hyperintensities

We segmented PVS in the centrum semiovale (CSO) and basal ganglia (BG) using T1-weighted and FLAIR images and a thoroughly validated PVS segmentation tool [45, 46]. The BG region of interest (ROI) encompassed the internal and external capsules, caudate, lentiform, and thalamic nuclei, while the CSO ROI included the remaining supratentorial white matter. Note that these ROIs do not precisely match anatomical structures, but we maintained the established nomenclature to ensure consistency with widely used visual rating techniques in the field [47]. A trained image analyst (JB) manually refined all PVS segmentation masks to reduce false positives.

We segmented white matter hyperintensities (WMH) using T1-weighted and FLAIR images and the AI-enhanced version of the Lesion Segmentation Toolbox (LST-AI). Using the USCLobes Atlas, we then measured WMH volumes across the entire brain, as well as across the cerebral lobes, corpus callosum, and internal and external capsules.

### Visit 6: physical fitness assessment

The physical fitness assessment comprised three measures to evaluate muscular fitness (visit 6). Handgrip strength was used to evaluate muscular strength (SAEHAN Professional), while the timed-up-and-go test served as a metric for assessing physical performance. Appendicular skeletal muscle mass (ASMM), as determined by bioelectrical impedance analysis (BIA 101 BIVA PRO), was utilized as a quantifiable indicator of muscle mass. Furthermore, blood pressure (systolic and diastolic) was measured in a supine position. The physical fitness assessment also included a cardiopulmonary fitness test (spiroergometry) for some participants (*n* = 137) to measure aerobic fitness, with VO_2_max as the main outcome, which was not analyzed here since not all participants underwent spiroergometry. Spiroergometry was discontinued in the course of the study due to limited personnel capacities. In addition to the general inclusion/exclusion criteria (see sample characteristics above), participants were excluded from the physical fitness assessment if their medical history revealed severe cardiac conditions (e.g., acute coronary syndrome, symptomatic severe aortic valve stenosis, congestive heart failure, or resting blood pressure > 180/100 mm Hg), acute orthopedic injuries (within the last 6 months), severe muscular diseases (e.g., active myositis), or severe endocrinological/metabolic disorders (e.g., morbid obesity [BMI > 30]). The median time interval between the physical fitness assessment and cognitive testing 2 was 214 days [(Q1, Q3): (123.5, 311.5) days].

### Questionnaires

Questionnaires were either handed out or mailed to participants to complete at home. These questionnaires included the 30-item version of the Geriatric Depression Scale (GDS) [48] and the State-Trait-Anxiety Inventory (STAI) [49–51] to assess mental health, the Pittsburgh Sleep Quality Index (PSQI) [52] and the Epworth Sleepiness Scale (ESS) [53] to measure sleep quality and daytime sleepiness, the 57-item Food Frequency Questionnaire (FFQ) [54] from the DEGS1 study (German Health Interview and Examination Survey for Adults) to assess diet, and the Silver-Santé version of the Lifetime of Experience Questionnaire (LEQ) [55, 56] to measure mental activity across the life course. Two additional questionnaires were administered by staff during visit 2, comprising the 36-item Short Form Health Survey (SF-36) [57, 58] to assess the general health status and the short form of the Freiburg questionnaire on physical activity (FFKA) [59]. All questionnaires were administered in their respective German versions.

We calculated the total scores of GDS, State and Trait Anxiety, ESS, PSQI, the early life and midlife LEQ non-specific subscales (see Supplementary Methods - Lifetime of Experience Questionnaire), and the physical and mental component summaries of the SF-36. The updated compendium of metabolic equivalent of task values [60] was used to calculate the metabolic equivalent of task per week for the domains of basic physical activity, leisure time physical activity and sports activities, thereafter called so, according to the manual. Alcohol consumption in grams per day was estimated based on the FFQ [61]. To further evaluate the FFQ, we used a previously published R script [62]. The script converts the frequency and amount of consumption of 53 foods in the last 4 weeks into units of grams per day for each food. Subsequently, we calculated the healthy eating index (HEI-DEGS) which was validated in the DEGS study [63], is based on the recommendations of the “Deutsche Gesellschaft für Ernährung” (DGE), and is tailored towards the 57-item version of the FFQ. In order to render the HEI-DEGS independent from alcohol consumption we excluded that component before calculating the total score.

Subject-wise mean imputation was used for the GDS and STAI subscales if 10% or less of the items were missing, for the LEQ subscales if 1 out of 7 items were missing, and for the SF-36 according to the manual. For the FFQ, isolated missing frequency data for a food were categorized as non-consumption, while missing quantity data were replaced by the population average. Missing items in the ESS, PSQI, and FFKA resulted in missing total scores.

### Statistical analyses

#### Cognitive age model

We applied a multivariate approach in the entire sample of older adults to predict cognitive age as previously described by Pezzoli and colleagues [22]. We estimated cognitive age based on the comprehensive cognitive testing data, which may reveal patterns of overall cognitive aging that individual neuropsychological measures do not readily detect [22]. We determined the “cognitive age gap” (CAG) as the difference between cognition-predicted age and chronological age. To estimate cognitive-predicted age, we used Partial Least Square (PLS) regression, a multivariate approach that reduces a set of predictor variables into latent variables (linear combinations of the original variables) by maximizing covariance with the response variables. PLS regression is well-suited to prediction tasks where predictor variables are highly correlated, such as cognitive tests, and allows for an easy interpretation of the loadings within each dimension, representing key features with respect to the prediction task.

More specifically, we used the plsregress.m function from the MATLAB (version 2024b) statistics and machine learning toolbox. Based on the results of an initial model with 5 components (as used in [22]) and the resulting “elbow plot,” we finally set the number of components to 3. We trained the model using the full cognitive data set of our study (acquired until 12/12/2024), that is, all subjects who had performed both cognitive testing visits. The chronological age at the cognitive testing visit was used as the response variable. The following tests/subtests were used as predictor variables: VLMT trials 1–5 (sum), VLMT immediate and delayed recall, VLMT recognition, RCFT immediate and delayed recall, RCFT recognition, SDMT, RWT food and jobs, FCRST free recall, WMS LM immediate recall, delayed recall, and recognition, verbal fluency animals (from CERAD), word list learning total and recall (from CERAD), and TMT B/A ratio. We included all subjects with no more than three missing scores out of the total 19 test scores. This led to the exclusion of two subjects with a final sample of 346 [age range: 60 to 94 years (*M* = 72, SD = 8), 166 (48%) female]. Of the 336 participants with complete data, 10 subjects had up to three missing test scores. The values from the VLMT (immediate, delayed recall and recognition), RWT foods and jobs, FCRST free immediate recall, WMS LM (I, II, and recognition), verbal fluency, word list learning and recall, and TMT B/A ratio (inverted to represent same directionality as other scores) were boxcox-transformed to reduce skewness of the data and get closer to a normal distribution (4 scores remained not normally distributed after the transformation). Next, test scores were z-transformed. Missing test scores were replaced by the mean z-score across all other tests for the 10 subjects with missing data.

We used tenfold cross-validation to predict cognitive age, by splitting the sample into 10 folds of random subsets (equally distributed across the entire age range), where 9 of the 10 folds are used to train the model and predictions are then made for the remaining fold. This process was repeated 10 times, with a different fold held out of the training each time (*n* = 34–35 subjects left out each time), in order to generate cognitive age predictions for all subjects. Next, we applied a statistical bias correction [64] to each individual’s predicted age to account for a frequently observed bias in age prediction found at the tails of the distribution, resulting in an overestimated age for younger adults and underestimated age for older adults [22, 65]. This is due to general statistical features of the regression analysis [66].

To assess prediction accuracy of chronological age by the model predicted age, we calculated the correlation between age and cognitive-predicted age (Pearson correlation coefficient *r*) and we report the average total variance explained (*R*^2^) by the cognitive scores across the 10 folds as well as average mean squared errors (output from the PLS model).

#### Brain age model

Similar to the estimation of cognitive age, we estimated brain age using the same PLS approach as above. While previous studies mainly used gray matter volume to estimate brain age, we focused on proxies of age-related diseases (Alzheimer’s and vascular disease) and neurodegeneration. We included the following predictors in our brain age model that are based on MRI and blood biomarkers: CSF volume, bilateral hippocampal volume, whole brain gray matter thickness, total WMH volume, number of PVS in BG and in CSO, plasma Aβ_1–42_/Aβ_1–40_ ratio, and pTau_217_. All volumes were adjusted for TICV obtained from SAMSEG. CSF volume, PVS counts, and plasma AD markers were boxcox-transformed, WMH volume was log-transformed. Hippocampal volume and gray matter thickness were inverted to represent atrophy, such that higher values would present more pathology for all measures. Otherwise, the PLS modelling was performed as above to predict brain age, but using 2 components (based on elbow plot and prediction accuracy of age). All subjects with processed MR data and plasma AD biomarkers were included [*n* = 261, age range: 60 to 94 years (*M* = 71, SD = 7), 120 female (46%)].

#### Derivation of latent components of lifestyle and general health

To characterize the lifestyle and health profiles in our cohort, a principal component analysis (PCA) was computed. We focused on 30 variables based on the medical history, questionnaires and blood markers related to bodily health. Conceptually, our 30 variables of lifestyle and general health span key domains previously related to cognitive aging and dementia risk and can be categorized into sleep (sleep quality, daytime sleepiness), diet (healthy eating index DEGS, alcohol), physical activity (basic physical activity, leisure time physical activity, sports activities), physical fitness (timed up-and-go test, appendicular skeletal muscle mass, handgrip strength), mental activity (education years, early life non-specific LEQ, midlife non-specific LEQ), cardiovascular risk factors [number of antihypertensive drugs, systolic blood pressure, diastolic blood pressure, HbA1c as a measure of blood sugar levels over the last 60–80 days, body mass index (BMI), pack years of smoking], mental health and functioning (SF-36 mental composite summary, geriatric depression, trait anxiety, state anxiety), and physical health and functioning (SF-36 physical composite summary, homocysteine, estimated glomerular filtration rate, albumin, leukocyte count, hemoglobin, platelet count).

Processing of lifestyle and general health data and statistical testing were carried out in R version 4.2.3 and RStudio version 2024.9.1.39. We first assessed the level of missing data for our *n* = 211 subjects and the aforementioned 30 features. Subject-wise more than 76.8% of subjects had complete data, with only 10.4% of subjects missing one feature, 11.4% of subjects missing two features, and 1.4% of subjects missing three or four features. Feature-wise 17 variables were complete for every subject, while 11 variables were missing for less than 6% of the subjects, and only two variables were missing for 11.4% of the subjects. Next, we assessed the univariate normality of our variables visually and with normality tests (MVN package). 25 of 30 variables were not normally distributed and were subsequently transformed with the functions of the bestNormalize package to approximate normal distribution as closely as possible. While for 10 out of these 25, no transformation was found to improve normality, for 7 out of the remaining 15 variables normality was achieved after transformation, while the other 8 variables were closer to normal distribution but were not yet normally distributed. Afterwards, three extreme outliers were identified with the identify_outliers function of the rstatix package which led to a listwise deletion of these subjects (one subject with very high daytime sleepiness, one with very high blood sugar levels and one with very high platelet count) and a final sample size of *n* = 208. In order to eliminate effects of sex and age in the variables of lifestyle and general health, we tested for age and sex effects first in conjunction, and if insignificant individually, with a general linear model and proceeded with the residual if the respective model was statistically significant. The rationale behind this is that we are primarily interested in the variance that goes beyond the effects that age and sex impose on some of these variables, for instance, more/less appendicular skeletal muscle mass than expected for the respective age and sex. Missing data was imputed with the missForest package, z-scaled afterwards and a PCA was conducted. To determine the number of principal components (PC) that should be retained we applied Horn’s parallel analysis (5000 iterations) with the paran package, which suggested seven components.

#### Statistical testing of association between lifestyle profiles, CAG, BAG, and APOE

First, we tested whether the derived lifestyle/health profiles related to CAG and BAG. For this purpose, we constructed two linear regression models with lm() in R consisting of the seven principal components as independent variables and CAG and BAG as dependent variables, respectively. Model requirements, namely linearity, normality, and homoscedasticity, were visually checked via diagnostic plots and met. As CAG and BAG were associated with sex, we also included sex as a covariate:

$$\begin{array}{c}CAG \sim PC1 + PC2 +PC3 +PC4 +PC5 +PC6 +PC7 +sex\\ BAG\sim PC1 + PC2 +PC3 +PC4 +PC5 +PC6 +PC7 +sex\end{array}$$

The alpha level for all statistical tests was defined as *p* < 0.05. To investigate the potential mediation of latent components of lifestyle and general health on CAG via BAG, we conducted a causal mediation analysis with the mediation package, running 10,000 Monte Carlo draws. The models used as input for the mediation analysis were:

$$\begin{array}{c}Model M: BAG \sim PC2 + sex\\ Model Y: CAG \sim BAG + PC2 +sex\end{array}$$

Second, we tested whether *APOE* ε4 carriers had a higher BAG or CAG than non-carriers using two two-sample *t*-tests (equal variances, one sided). We report Cohen’s *d* as effect size.

## Results

### Sample characteristics

We recruited and successfully included a total of 348 participants between 2021 and 2024, among which 206 individuals had derived lifestyle/health profiles and CAG and 171 participants had BAG estimates (after preprocessing; see Supplementary Fig. 1 for flow chart). An overview of main demographics and key variables is given in Table 1 for the CAG and BAG samples. Notably a high number of participants in our sample were older than 79 years (*n* = 44) and participants were generally highly educated (also see Supplementary Fig. 2). The additional *n* = 35 who were in the CAG sample but not in the BAG subsample were significantly older but did not differ in other characteristics compared to the rest of the sample (Supplementary Table 1).

**Table 1:** Sample characteristics

	**CAG sample (*n* = 206)**	**BAG subsample (*n* = 171)**
**Age (years)**		
Mean (*SD*), [Min, Max]	70.8 (7.4), [60.3, 88.9]	70.2 (6.9), [60.3, 87.1]
**Sex**		
Female/male (%)	96/110 (47/53%)	78/93 (46/54%)
**Education (years)**		
Mean (*SD*), [Min, Max]	14.8 (2.4), [10.0, 20.0]	14.9 (2.3), [11.0, 20.0]
**MMSE z-value**		
Mean (*SD*), [Min, Max]	0.02 (0.87), [−2.51, 1.77]	0.04 (0.87), [−2.19, 1.77]
**CERAD + z-value**		
Mean (*SD*), [Min, Max]	0.48 (0.38), [−0.42, 1.61]	0.48 (0.39), [−0.42, 1.61]
**GDS-30**		
Mean (*SD*), [Min, Max]	4.0 (4.2), [0.0, 24.0]	4.0 (4.3), [0.0, 24.0]
***APOE* ε4**		
Carrier/non-carrier (%)	48/156 (23/76%)*	40/130 (23/76%)^†^
**Aβ**_**1–42**_**/Aβ**_**1–40**_		
Mean (*SD*), [Min, Max]	0.090 (0.011), [0.057, 0.113]^†^	0.091 (0.011), [0.057, 0.113]
**pTau**_**217**_** (pg/ml)**		
Mean (*SD*), [Min, Max]	0.130 (0.091), [0.047, 0.684]^†^	0.125 (0.082), [0.047, 0.678]

*APOE* ε4 “carrier” represents individuals with at least one ε4 allele; one subject had a z-score of − 2.51 with a raw score of 25 in the MMSE, but was cleared by the Cognition Expert Panel based on otherwise normal cognitive test results; *CAG*, cognitive age gap; *BAG*, brain age gap; *MMSE*, Mini Mental State Examination; *CERAD*, Consortium to Establish a Registry for Alzheimer’s disease; *GDS*, Geriatric Depression Scale; *APOE*, Apolipoprotein E; *Aβ*, amyloid-beta; *pTau*, phospho-tau; *data of 2 subjects missing; ^†^data of 1 subject missing

### Prediction of cognitive and brain age

As shown in Supplementary Figures S3 and S4, age was moderately correlated with cognitive scores (*r*-values between − 0.17 and − 0.48 for 17 out of 19 scores) as well as brain-pathology related scores (*r*-values between 0.19 and 0.57 for 8 out 9 variables). Cognitive age and brain age were predicted by PLS regression using 10 folds/runs, where the PLS model was estimated based on 90% of the data (participants) and applied on the 10% left-out data (participants) to predict age. The cumulative percentage variance explained in age (average across the 10 folds) by the 19 cognitive predictor variables was 36% (SD = 1.6). Regarding brain age, the cumulative percentage variance explained in age by the brain predictor variables was 55% (SD = 1.8).

**Fig. 2:**
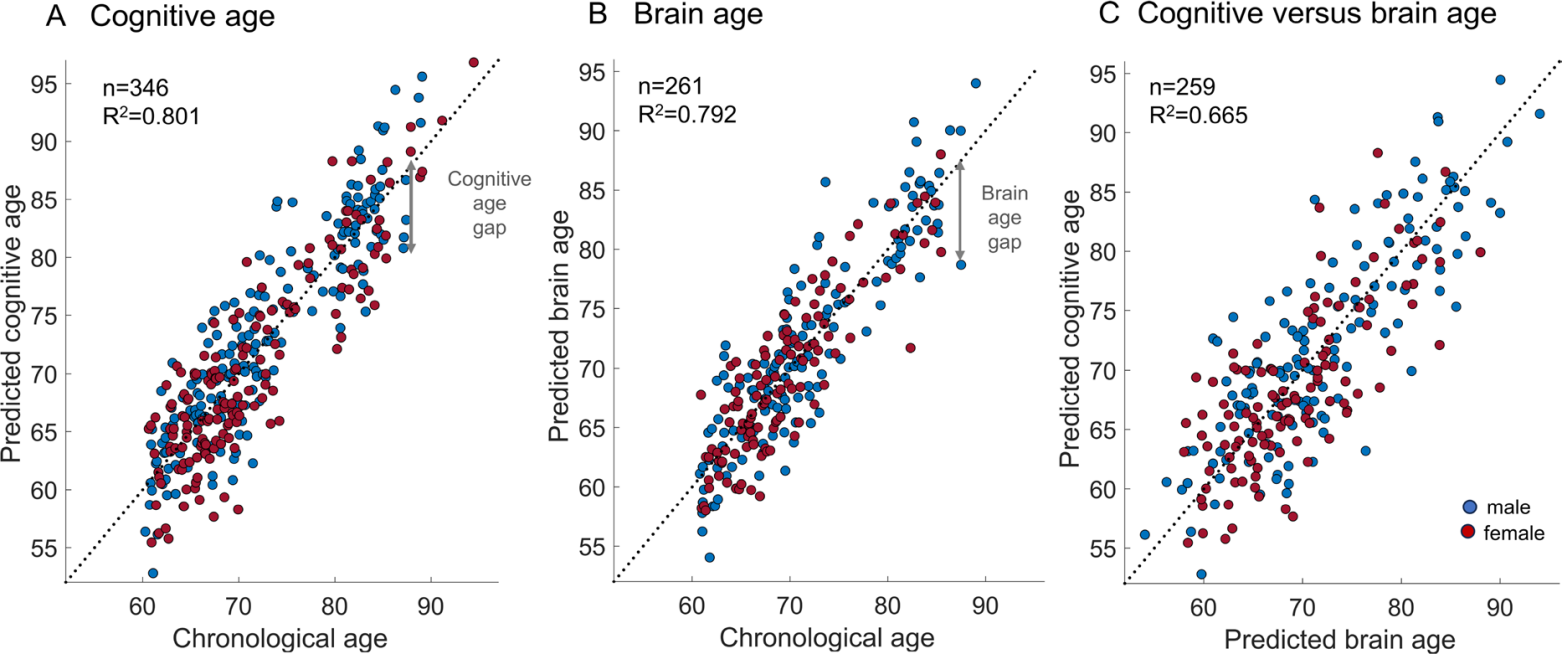
Cognitive and brain age. **A** Cognitive age and **B** brain age were predicted from partial least squared (PLS) regression models based on cognitive scores and brain-pathology-related markers (atrophy proxies, white matter hyperintensities, perivascular spaces, and AD biomarkers), respectively. Scatterplots show predicted age after age-bias correction by chronological age (see Supplementary Figures S4B and S5B for results before bias correction). The dashed lines are lines of identity (y = x) where predicted age = chronological age. *R*^2^ value refers to the correlation strength of chronological and predicted cognitive or brain age (after bias correction). Arrows denote the difference between chronological and predicted cognitive (**A**) and brain age (**B**). **C** Correlation of predicted brain age and predicted cognitive age

The variance explained by chronological age in predicted cognitive age (for unseen subjects) before bias correction was *R*^2^ = 0.307 (Supplementary Figure S5) and after age-bias correction *R*^2^ = 0.801 (Fig. 2A). The variance explained by chronological age in predicted brain age before age-bias correction was *R*^2^ = 0.519 (Supplementary Figure S6) and after bias correction *R*^2^ = 0.792 (Fig. 2B). Mean absolute error was 3.2 (SD = 2.4) years for cognitive age and 2.8 (SD = 2.2) years for brain age after bias correction.

CAG and BAG scores were obtained by subtracting the chronological age from the corresponding predicted age after correction for the frequently observed statistical bias in age prediction that was also evident in our data (Supplementary Figures S5 and S6). Consequently, negative CAG or BAG values denote younger cognitive or brain age than expected (as the arrow illustrates for one subject in Fig. 2). While CAG values were significantly higher in males compared to females (*M*_*male*_ = 0.63 versus *M*_*female*_ = − 0.68, *CI* [0.5 2.1], *p* = 0.002), BAG values did not differ by sex (*M*_*male*_ = 0.20 versus *M*_*female*_ = − 0.23, *CI* [−0.4 1.3], *p* = 0.32). BAG and CAG were significantly, weakly correlated (*r* = 0.19, *p* = 0.002).

### Latent components of lifestyle and general health

**Fig. 3:**
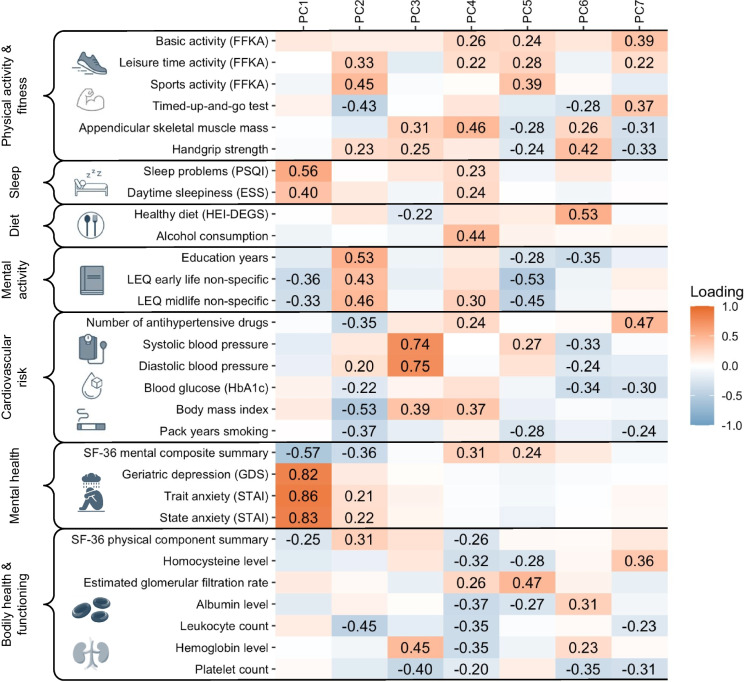
Loadings of the 30 lifestyle/health variables on the seven principal components that were retained with Horn’s parallel analysis. The first five principal components were named *Low Mental Health* (PC1), *Active Life* (PC2), *Acute High Blood Pressure* (PC3), *Robust Physique & High Alcohol Consumption* (PC4), *Mentally Inactive & Physically Active* (PC5) according to the main factors loading on the principal components, respectively. Display of factor loadings was thresholded at − 0.2/+ 0.2

We ran a PCA on thirty variables quantifying aspects of lifestyle and general health. Applying Horn’s parallel criterion yielded seven components which should be retained (Supplementary Figure S7). These seven PCs explained 48.8% of the total variance (Supplementary Figure S8; factor loadings in Fig. 3). We then named the principal components according to variables with the greatest loadings: The first principal component (PC1 *Low Mental Health*; 11.6% of explained variance) was mainly characterized by low mental health as indicated by GDS, STAI, SF-36 mental composite summary, accompanied by sleep problems as indicated by PSQI and ESS, and low mental stimulation throughout life as manifested in the LEQ subscales. The second principal component (PC2 *Active Life*; 8.6% of explained variance) was very broad, reflecting high education and mental activity throughout life (LEQ subscales), high physical activity and fitness (free time, sports activities, timed-up-and-go-test, handgrip strength), a low cardiovascular risk profile (BMI, HbA1c, pack years smoking, number of antihypertensive drugs; however, a subtle tendency for higher diastolic blood pressure), and low leukocyte counts. Interestingly, this component was also characterized by reduced mental health, albeit more subtly than PC1. Principal component three (PC3 *High Blood Pressure*; 7.2% of explained variance) showed mainly high loads of acute high blood pressure and accompanying high hemoglobin. It also displayed high muscular strength (ASMM and handgrip strength), a less healthy diet (HEI-DEGS), high BMI and lower platelet counts. The fourth principal component (PC4 *Robust Physique & High Alcohol Consumption*; 6.2% of explained variance) captured high alcohol consumption, accompanying cardiovascular risk indicated by high BMI and a high number of antihypertensive drugs, low blood cell counts, low physical health (SF-36 physical component summary), and sleep problems (PSQI, ESS). This was opposed by a muscular body composition (ASMM) and low-intensity physical activity (basic and leisure time), good mental health (SF-36 mental composite summary) and mental activity in midlife (LEQ subscale), and rather good kidney function (eGFR, albumin, homocysteine). The fifth principal component (PC5 *Mentally Inactive & Physically Active*; 5.9% of explained variance) was mainly characterized by low mental activity throughout life (LEQ subscales) and low education, high physical activity in general (basic, leisure time, sports activities), but low muscular strength (ASMM, handgrip strength). It also reflected low pack years of smoking, but high systolic blood pressure, high mental health (SF-36 mental component summary) and good kidney function (eGFR, albumin, homocysteine). Since principal components six (4.8% of explained variance) and seven (4.5% of explained variance) were only slightly above chance level (i.e., higher eigenvalues than derived by Monte Carlo simulated data), we will not describe them in detail at this point.

### Associations of the latent components with CAG and BAG

**Fig. 4:**
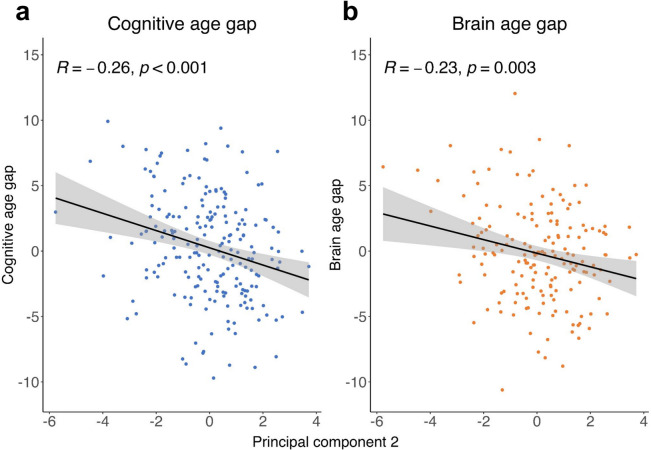
Linear regression of principal component (PC) 2 *Active Life* against cognitive age gap (left plot) and brain age gap (right plot). PC2 was significantly associated with both cognitive age gap (**A**) and brain age gap (**B**). The black line represents the regression line with its 95% confidence interval (gray area), while dots represent individual data points. *p* < 0.05 was considered statistically significant; correlation coefficient, *R*; please note that raw values are plotted

The seven components derived from the lifestyle and general health data were included in a general linear model to predict CAG. As CAG was associated with sex, we also included sex as a covariate in the model. Three principal components were significantly associated with CAG (Table 2), namely, PC1 *Low Mental Health* was positively associated (*β* = 0.36, *CI* [0.08, 0.63], *p* = 0.011), PC2 *Active Life* was negatively associated (Fig. 4; *β* = − 0.66, *CI* [− 0.98, − 0.34], *p* < 0.001), and PC5 *Mentally Inactive & Physically Active* was positively associated (*β* = 0.51, *CI* [0.13, 0.90], *p* = 0.003). Collectively, these results show prominent effects of good mental health (PC1), an active lifestyle, particularly characterized by mental activities (PC1, PC2, PC5), physical activity and fitness (PC2, PC5), and rather low vascular risk (PC2) for having a low CAG, i.e., younger predicted cognitive than chronological age.

**Table 2:** Results of the linear regressions of CAG and BAG on the principal components

	**Cognitive age gap**			**Brain age gap**		
*Predictors*	*Estimates*	*CI*	*p*	*Estimates*	*CI*	*p*
(Intercept)	* − 0.68*	* − 1.43, 0.07*	*0.075*	* − 0.74*	* − 1.55–0.07*	*0.074*
PC1	**0.36**	**0.08, 0.63**	**0.011**	* − *0.00	* − *0.29–0.28	0.993
PC2	–**0.66**	* − * **0.98,** **0.34**	** < 0.001**	* − ***0.52**	* − ***0.86 to** − **0.18**	**0.003**
PC3	0.15	* − *0.20, 0.50	0.403	* − *0.06	* − *0.43–0.32	0.770
PC4	* − *0.24	* − *0.62, 0.13	0.197	*0.35*	* − 0.03–0.73*	*0.073*
PC5	**0.51**	**0.13, 0.90**	**0.009**	* − *0.04	* − *0.45–0.36	0.833
PC6	0.18	* − *0.25, 0.61	0.411	* − *0.23	* − *0.68–0.22	0.323
PC7	0.16	* − *0.28, 0.60	0.474	* − *0.08	* − *0.54–0.39	0.750
Sex [male]	**1.55**	**0.53, 2.58**	**0.003**	**1.10**	**0.00–2.20**	**0.050**
	Observations	*n* = 206		Observations	*n* = 171	
	*R*^2^ = 0.179			*R*^2^ = 0.097		

Results of linear regression for cognitive age gap (*n* = 206) and brain age gap (*n* = 171) as dependent variables each. Predictors included the lifestyle/health principal components: *Low Mental Health (PC1)*, *Active Life (PC2)*, *Acute High Blood Pressure (PC3)*, *Robust Physique & High Alcohol Consumption (PC4)*, *Mentally Inactive & Physically Active (PC5), PC6, and PC7, as well as sex.* Bold font indicates statistical significance (*p* < 0.05); font in italics marks trends (*p* < 0.1); 95% confidence interval, *CI*; total variance explained, *R*^2^; *PC*, principal component

Analogously, we built a general linear model including the seven principal components and sex as predictors for BAG. This model only yielded a significant negative association with BAG for PC2 (Fig. 4; *β* = − 0.52, *CI* [− 0.86, − 0.18], *p* = 0.003) and a trend for a positive association of PC4 (*β* = 0.35, *CI* [− 0.03, 0.73], *p* = 0.073) with BAG. The relation between higher PC2 and lower BAG, i.e., younger predicted brain age than chronological age, displays the effect of a mentally and physically active lifestyle with low cardiovascular risk on good brain health. The trend observed for PC4 indicates a particular prominence of low cardiovascular risk in this association, as indicated by the positive loadings of alcohol consumption, antihypertensive medications and BMI on PC4.

Sensitivity analyses that included education years as an additional covariate in the regression model weakened the associations of the principal components with CAG and BAG, although the associations remained significant (Supplementary Table 2). Finally, we also note that sex was significantly associated with CAG and on a trend level with BAG, with a lower cognitive and brain age gap observed in females (Table 2).

**Fig. 5:**
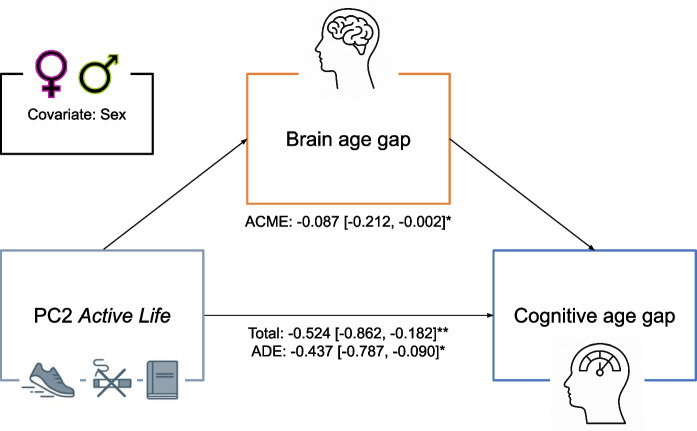
Causal mediation analysis of PC2 *Active Life* via brain age gap on cognitive age gap with sex as covariate. A significant portion of the total effect of principal component (PC) 2 on cognitive age gap was mediated by brain age gap. However, the direct effect remained significant, indicating a partial mediation. Total effect of PC2 on cognitive age gap, total; average causal mediation effect, ACME; average direct effect (after taking the mediation effect into account), ADE; *p < 0.05; **p < 0.01; ***p < 0.001; 95% confidence in square brackets

Due to the overlapping association of PC2 with CAG and BAG, we tested post-hoc whether the effect of PC2 on CAG was mediated by its effect on BAG. Accordingly, we ran a causal mediation analysis, again including sex as a covariate (Fig. 5). This causal mediation analysis evidenced a significant negative total effect of PC2 on CAG (*β* = − 0.524, *CI* [− 0.862, − 0.182], *p* = 0.002) which was reduced, but still significant (*β* = − 0.437, *CI* [− 0.787, − 0.090], *p* = 0.016), upon inclusion of BAG as a mediator of this relationship. The mediation of PC2 via BAG on CAG was significant (*β* = − 0.087, *CI* [− 0.212, − 0.002], *p* = 0.039), explaining a proportion of 16.6% (*CI* [0.4%, 58.3%]) of the effect of PC2 on CAG being explained by its effect on BAG. In summary, this partial mediation suggests that the effect of the *Active Life* profile PC2 on younger (than expected) cognitive age may partially be explained by a younger (than expected) brain age.

### Associations of APOE genotype with CAG and BAG

Two sample *t*-tests showed that *APOE* ε4 carriers had significantly higher BAG (*t* = 1.66, *df* = 233, *p* = 0.049, *d* = 0.25; see Supplementary Table 3 for sample characteristics), but not a higher CAG (*t* = 0.78, *df* = 299, *p* = 0.217, *d* = 0.11; see Supplementary Table 4 for sample characteristics) than non-carriers, i.e., *APOE* ε4 carriers had significantly older brain age than expected compared to non-carriers.

## Discussion

In the current study, we examined how latent lifestyle and health profiles were associated with brain and cognitive health in a deeply phenotyped cognitively unimpaired aging cohort. In particular, we combined extensive, multifaceted data on lifestyle and health, as well as brain and cognitive imaging indices, to gain insight into age-related cognitive resilience. Our study revealed that a younger-than-expected cognitive and brain age are convergently linked to an active lifestyle profile characterized by high physical fitness, high mental stimulation, and low cardiovascular risk. Moreover, older-than-expected cognitive age, but not brain age, was related to lifestyle profiles characterized by low mental stimulation. Furthermore, the *APOE* ε4 genotype, a genetic risk factor for AD, was linked to older-than-expected brain age, but not cognitive age. Our study suggests that a mentally and physically active lifestyle is linked to successful cognitive aging, partially explained by avoidance of pathological brain changes. Additionally, engagement in mental leisure activities may promote cognitive resilience, independent of brain pathology, potentially via reserve mechanisms.

Modifiable risk factors account for around 45% of population variability in dementia risk [67], but which lifestyle and health profiles relate to successful brain and cognitive aging, is less clear. By using an unsupervised data-driven approach such as PCA, we were able to first describe latent profiles of lifestyle behaviors and health status in our cognitively unimpaired aging cohort, by capturing naturally occurring covariance structures, in our case in and between different lifestyle and health domains. Previous population-based studies have shown that healthy lifestyle behaviors (e.g., physical activity and a healthy diet) or the prevalence of risk factors (e.g., smoking and drinking) tend to co-occur [68–70]. For instance, a recent study used latent profile analysis on four lifestyle behaviors (physical activity, cognitive activity, healthy diet, and social activity) in a cohort of older adults [19]. The study revealed three profiles characterized by high, moderate and low engagement in all four lifestyle behaviors, with those who had high engagement in one lifestyle behavior showing similar levels in the other behaviors. Moderate and low engagement groups showed faster decline in global cognition in that study. Similarly, in our first principal components we observed mostly expected associations across domains. In PC1 mental health problems were linked to sleep problems and a less stimulating life, in PC2 a healthy lifestyle as indicated by physical and mental activity was related to lower cardiovascular risk and better bodily function, and in PC3 acute blood pressure was associated with an unhealthy diet and high BMI. However, we also observed lifestyle and health profiles that combined conceptually contrasting domains, such as higher physical activity and muscle strength in association with higher cardiovascular risk in PC4, or profiles combining domains that are intuitively conceptually unrelated, such as a physically stimulating but mentally unstimulating lifestyle in PC5. Thus, by using PCA, we were able to extract and describe the most prominent lifestyle and health profiles that are present in our sample with different potential combinations of objective and subjective mental and physical health.

Strong associations have been consistently found between cardiovascular risk factors, in particular alcohol consumption and smoking history, and indices of brain aging such as gray matter volume/thickness, white matter microstructure, and BAG for middle-aged and older individuals [71–77], consistent with our findings. Of note, most of the cardiovascular risk in our study was captured by a negative factor loading on PC2, while alcohol consumption loaded almost exclusively on PC4. Interestingly, this component was related to older-than-expected brain age at trend level, and examining the individual correlation of alcohol consumption with BAG (Supplementary Figure S9) highlighted its role as the strongest individual predictor. While our results suggest that modifiable lifestyle behaviors, such as drinking and smoking, promote pathological brain changes, we also found that a genetic risk factor for AD and brain aging in general [32], namely *APOE* ε4, may explain a higher-than-expected brain age, possibly by negatively affecting brain maturation [78] or rendering the brain more susceptible to cortical atrophy [79]. Of note, we included plasma levels of pTau and Aβ in the estimation of BAG, which may have driven this relationship (see Supplementary Table 4).

With respect to cognitive age, it is striking that the three principal components on which early and midlife experience loaded were consistently associated with CAG, suggesting that a more mentally stimulating life is associated with a lower CAG. Higher LEQ scores captured more engagement in various activities, such as visiting family/friends, playing a musical instrument, artistic pastimes, reading, learning/speaking a second language, and traveling. A post hoc correlation analysis confirmed that the LEQ subscales and education years were most strongly correlated with CAG (Supplementary Figure S9) in line with a prior study finding that higher education and premorbid intelligence quotient were associated with lower CAG [24] and the role of leisure activities in preventing cognitive decline [80]. A recent study on “SuperAging” also reported musical background as a differentiating factor, where individuals in their 80s with superior memory performance were more likely to have a formal or amateur musical background than typical older adults [81]. Interestingly, in our study, the association between LEQ and CAG remained relatively high even after correcting for education years (Supplementary Figure S10), suggesting that the relationship is not only driven by differences in socioeconomic status and premorbid intellectual abilities but might actually capture beneficial effects of a mentally stimulating lifestyle that may manifest as cognitive resilience.

Our *Active Life* component (PC2) was the only lifestyle profile associated with both younger-than-expected cognitive and brain age. Considering how the variables prominently loading on this latent component were individually associated with CAG and BAG (Supplementary Figure S9), there seems to be a partial dissociation between lifestyle/health domains which are related to CAG versus BAG. In particular, cardiovascular risk and physical fitness were more strongly associated with BAG, whereas a mentally stimulating life and education were more strongly associated with CAG, consistent with a previous study [24]. However, as evidenced by the partial mediation of PC2 on CAG via BAG, shared associations existed, for instance, as indicated by the beneficial association with handgrip strength. Furthermore, the direction of the association between lifestyle and health variables expressed in PC2 and BAG compared to CAG was consistent for several variables, for instance, years of education, early life experience, number of antihypertensive drugs, and BMI. Thus, despite the fact that some of these associations were weak, this suggests a common basis for factors of brain maintenance and cognitive resilience that may be captured by the *Active Life* profile. In line with this, in a prior population-based study, the effect of modifiable cardiovascular risk factors on cognitive functioning was mediated by BAG [82].

The study has several limitations. First, we used cross-sectional data to estimate BAG and CAG, while brain maintenance and cognitive resilience should be ultimately studied longitudinally. This may partially explain the relatively weak association between BAG and CAG (*r* ~ 0.2), which is consistent with a previous study showing no significant association between both measures [24]. The dissociation between these measures could also be due to cognitive resilience despite age-related pathology or inter-individual differences in cognitive ability that are not directly tied to cognitive decline [2, 83]. Additionally, BAG, constructed with brain indices like WMH and AD biomarkers that progress over time may better capture age-related pathological changes than CAG captures cognitive decline. Longitudinal data collection in our ongoing study will allow for future analyses that account for brain and cognitive aging trajectories. Another limitation is the reliance on questionnaire-based lifestyle measures, which are prone to subjective bias. While we included objective measures like physical fitness assessments and blood tests, questionnaires that focus on short-term behaviors may reflect seasonal trends and obscure the long-term impact of lifestyle factors. Furthermore, variability in the time between visits, particularly between cognitive testing (visit 2) and fitness assessment (visit 6), may contribute to dissociation of lifestyle trajectories from brain and cognitive outcomes.

Furthermore, our cohort consists of primarily Caucasian, highly educated and mainly East German participants with limited ethnic diversity. There is also a gap in the age distribution, particularly in the 75–80 age group. While we are addressing this with ongoing data collection, the findings may not be directly applicable to other aging populations. For instance, a recent study that used canonical correlation analysis to study latent factors of lifestyle and brain structure in two independent samples, observed differential associations [76]. While in the Danish cohort, strongest contributions were seen for smoking and depressive symptoms, in the British cohort high blood pressure was most strongly contributing to low brain outcomes. Additionally, sex-specific associations between lifestyle and brain health were not explored, as we adjusted for sex and age before conducting principal component analysis. Sex has been shown to moderate various lifestyle-brain relationships [84] and complex sex and gender differences in cognitive resilience to aging and AD exist [85]. Future analyses will address sex-specific resilience to aging and AD, as complex sex and gender differences in cognitive aging are well-documented.

## Conclusion

While we observed several multifaceted health and lifestyle profiles in our cohort of older adults, a lifestyle characterized by high levels of mental stimulation throughout life, low cardiovascular risk, and high physical activity was jointly linked to a younger-than-expected brain and cognitive age. In particular, cardiovascular risk, most prominently current alcohol consumption and smoking history, were related to older-than-expected brain age, while mental stimulation in early and midlife and education years were related to younger-than-expected cognitive age. These findings suggest that such a mentally and physically active lifestyle may promote resistance against age-related brain pathological changes including brain atrophy, cerebrovascular and Alzheimer’s related pathology (brain maintenance), and cognitive resilience. Given the globally growing aging population and the consequently growing burden of cognitive disorders, our findings have implications for public health strategies aimed at promoting successful aging and delaying the onset of dementia. Future longitudinal studies are essential to further validate these associations and to explore the underlying mechanisms driving cognitive resilience in aging.

## Supplementary Information

Below is the link to the electronic supplementary material.

Supplementary file1 (DOCX 2.49 MB)

## Data Availability

The datasets generated and/or analysed during the current study are not publicly available due to the inclusion of sensitive participant information and privacy concerns but are available from the corresponding author on reasonable request.
