# Supplementary material for: A physically and mentally active lifestyle relates to younger brain and cognitive age
Source: GeroScience. 2025 Jul 7;48(2):1853–73. doi: 10.1007/s11357-025-01764-w (PMC12972277; doi:10.1007/s11357-025-01764-w)
Supplement: Supplementary file 1 — Supplementary file1 (DOCX 2.49 MB) [file 11357_2025_1764_MOESM1_ESM.docx]

A physically and mentally active lifestyle relates to younger brain and cognitive age

in GeroScience

Niklas Behrenbruch^1,2,3*^, Svenja Schwarck^1,3*^, Beate Schumann-Werner^1,3^, Eóin N. Molloy^1,4^, Berta Garcia-Garcia^1,4^ , Anne Hochkeppler^1,3^, Larissa Fischer^1,5^ , Anna-Therese Büchel^1^, Enise I. Incesoy^1^ , Jose Bernal^1,3,6^, Niklas Vockert^1^, Patrick Müller^1,7^, Gusalija Behnisch^8^, Bárbara Morgado^9^, Hermann Esselmann^9^, Constanze I. Seidenbecher^8,10^, Björn H. Schott^8,9,11^, Henryk Barthel^12^, Osama Sabri^12^, Jens Wiltfang^9,11,13^, Michael C. Kreissl^1,4^, Emrah Düzel^1,3,10^, and Anne Maass^1,2,10^

* These authors contributed equally

1 German Center for Neurodegenerative Diseases (DZNE), Magdeburg, 39120, Germany

2 Faculty of Natural Sciences, Otto von Guericke University Magdeburg, Magdeburg, 39106, Germany

3 Institute of Cognitive Neurology and Dementia Research (IKND), Otto von Guericke University Magdeburg, Magdeburg, 39120, Germany

4 Division of Nuclear Medicine, Department of Radiology & Nuclear Medicine, Faculty of Medicine, Otto von Guericke University Magdeburg, Magdeburg, 39120, Germany

5 Department of Neurobiology and Behavior, University of California, Irvine, Irvine, 92697, USA

6 Centre for Clinical Brain Sciences, The University of Edinburgh, Edinburgh, EH16 4SB, UK

7 University Hospital Magdeburg, Division of Cardiology and Angiology, Magdeburg, 39120, Germany

8 Leibniz Institute for Neurobiology (LIN), Magdeburg, 39118, Germany

9 Department for Psychiatry and Psychotherapy, University Medical Center Göttingen

(UMG), Göttingen, 37975, Germany

10 Center for Behavioral Brain Sciences (CBBS), Magdeburg, Germany

11 German Center for Neurodegenerative Diseases (DZNE), Göttingen, 39106, Germany

12 Department of Nuclear Medicine, University Hospital Leipzig, Leipzig, 04103, Germany

13 Neurosciences & Signaling Group, Institute of Biomedicine (iBiMED), Department of Medical Sciences, University of Aveiro, Aveiro, 3810-193 Portugal

**Corresponding author:**

Anne Maass, Ph.D.
anne.maass@dzne.de

+49 391 67-24620

German Center for Neurodegenerative Diseases

Leipziger Str. 44, Haus 64
39120 Magdeburg

# Supplementary Material

## Supplementary Figures

##
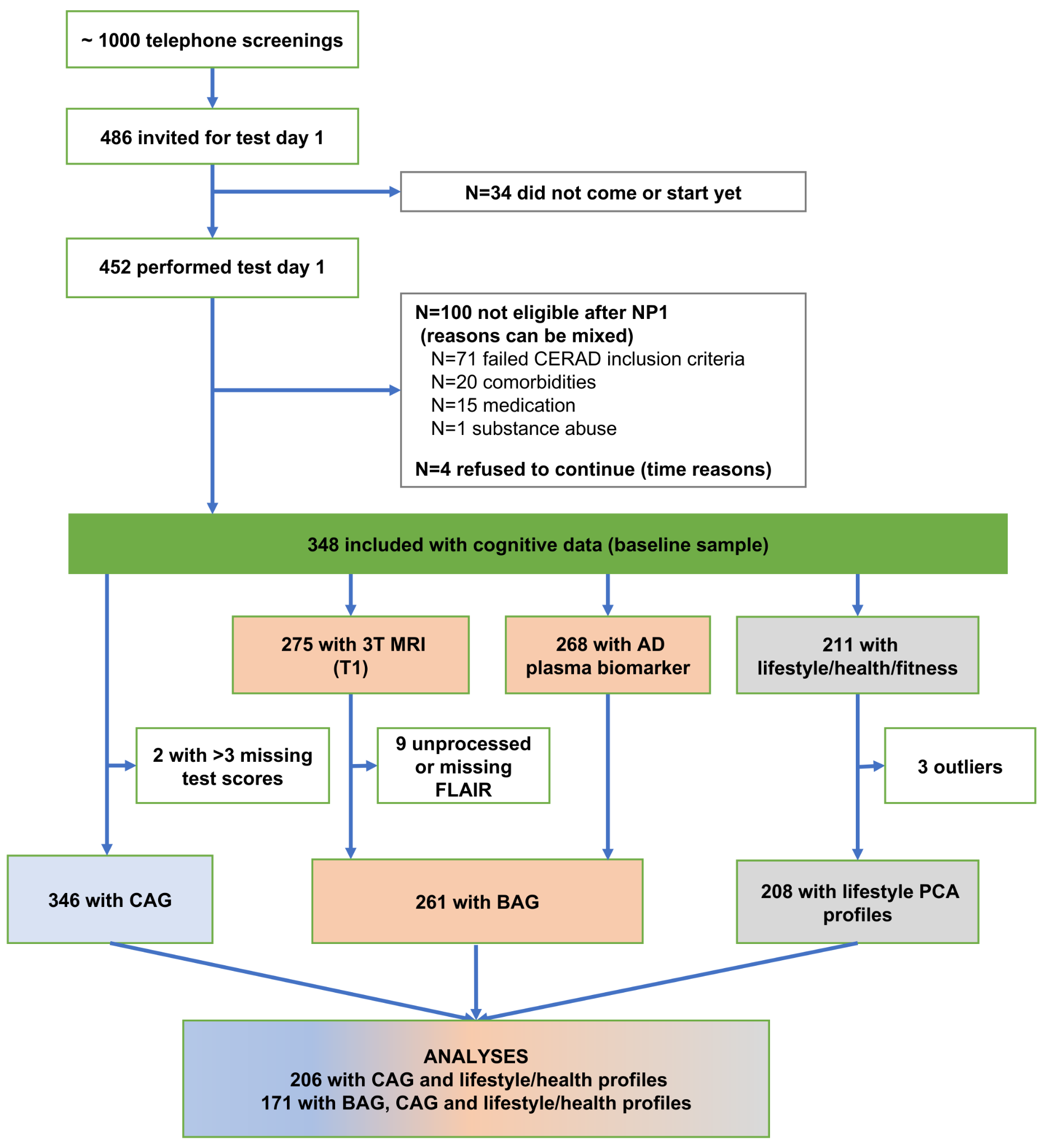
Supplementary Fig. S1 - Flow chart describing the selection process of participants. Alzheimer’s disease, AD; brain age gap, BAG; cognitive age gap, CAG; fluid-attenuated inversion recovery (used for estimation of white matter hyperintensities and enlarged perivascular spaces), FLAIR; principal component analysis, PCA


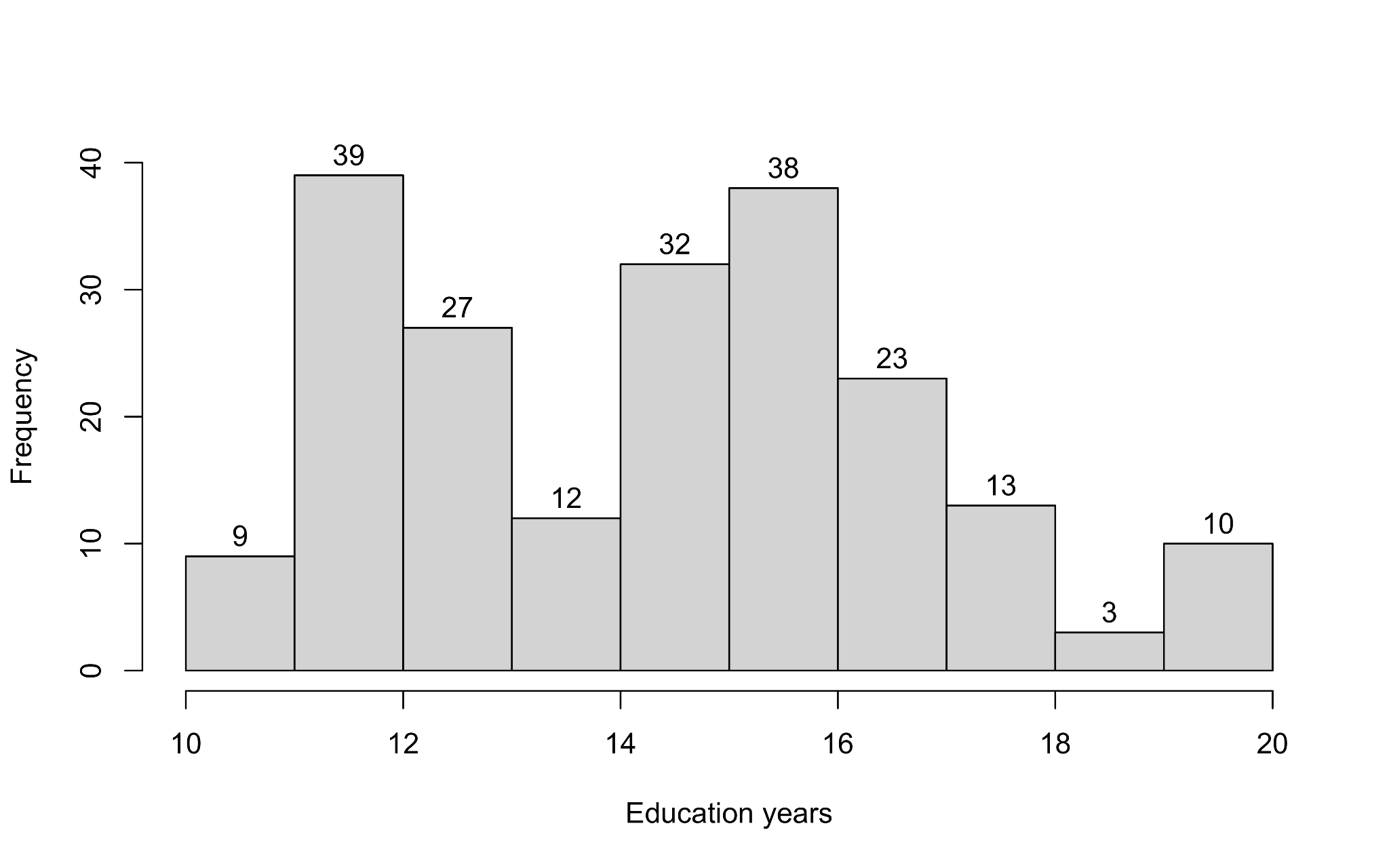


**Supplementary Fig. S2** - Histogram showing the distribution of education years in the sample


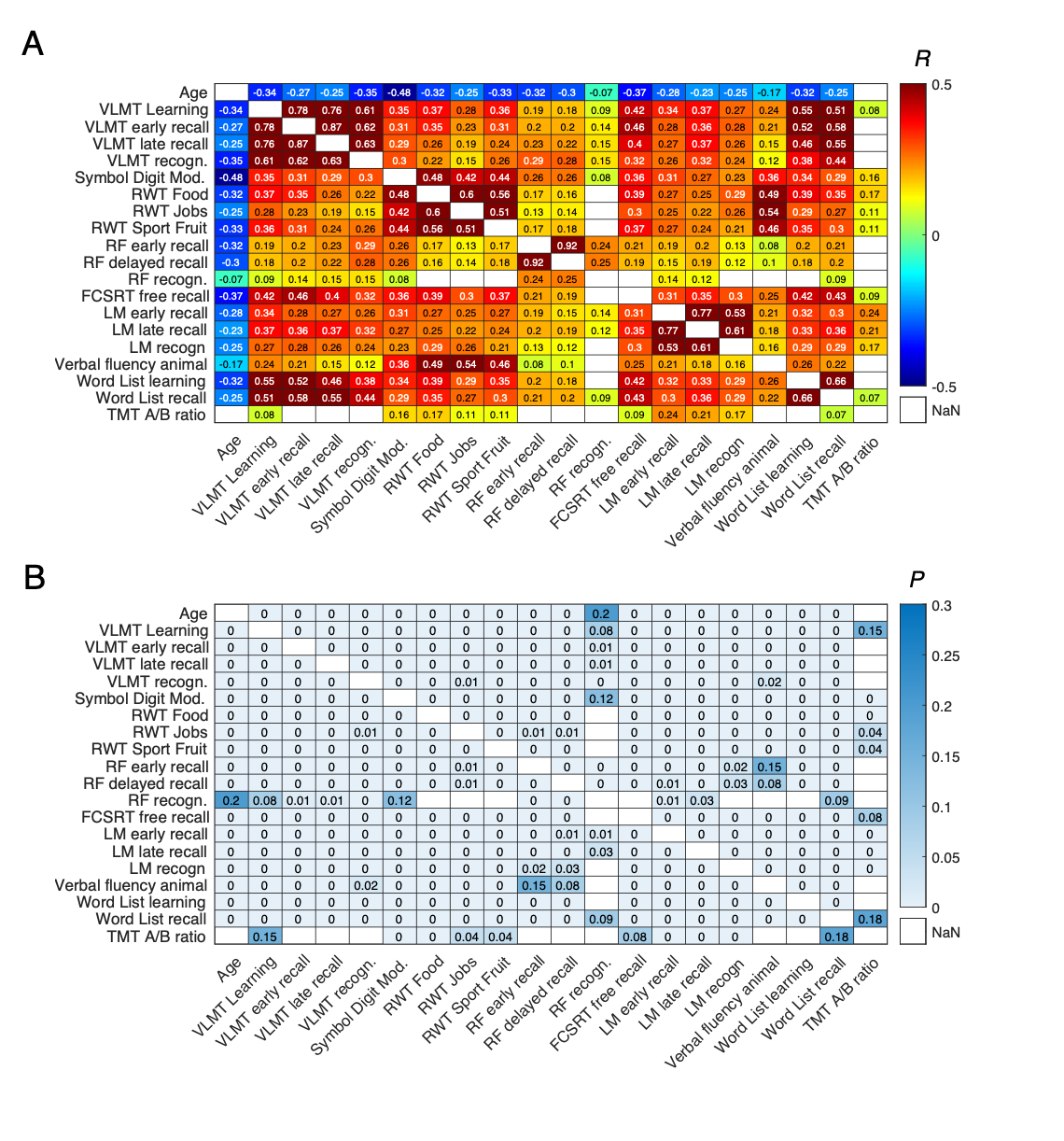


**Supplementary Fig. S3** - Correlation matrix of cognitive scores and age. Correlation strength *R* (A) and uncorrected *p*-values (B) of bivariate Pearson correlations between age and all cognitive scores that were included in the partial least squares regression to predict (cognitive) age. For display purposes only, correlations with *p*<0.25 are shown. All test scores were negatively correlated with age except for Rey Figure recognition and TMT A/B ratio (ratio was inverted such that higher values represent worse performance). Verbal Learning and Memory Test, VLMT; Modalities, Mod.; German Regensburger Wortflüssigkeitstest, RWT; Rey Figure, RF; Free and Cued Selective Reminding Test, FCRST; Logical Memory, LM


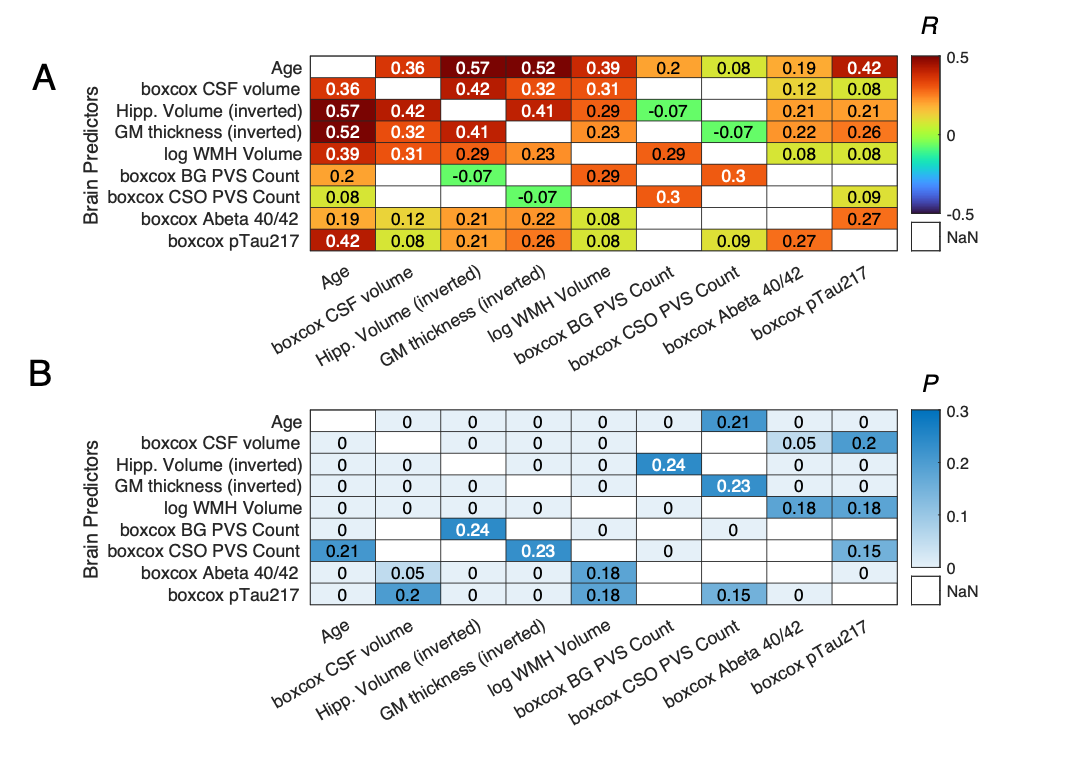


**Supplementary Fig. S4** - Correlation matrix of brain (pathology) scores and age. Correlation strength *R* (A) and uncorrected *p*-values (B) of bivariate Pearson correlations between age and all brain-health related scores (based on MR imaging and blood markers) that were included in the partial least squares regression to predict (brain) age. For display purposes only, correlations with *p*<0.25 are shown. All predictors of brain pathology were positively correlated with age except for CSO PVS. Hippocampal volume and gray matter (GM) thickness values were inverted such that higher values represent more atrophy. Volumes of hippocampus, CSF, WMH and PVS counts were adjusted for intracranial volumes. Basal Ganglia, BG; cerebrospinal fluid, CSF; centrum semiovale, CSO; perivascular spaces, PVS; white matter hyperintensities, WMH


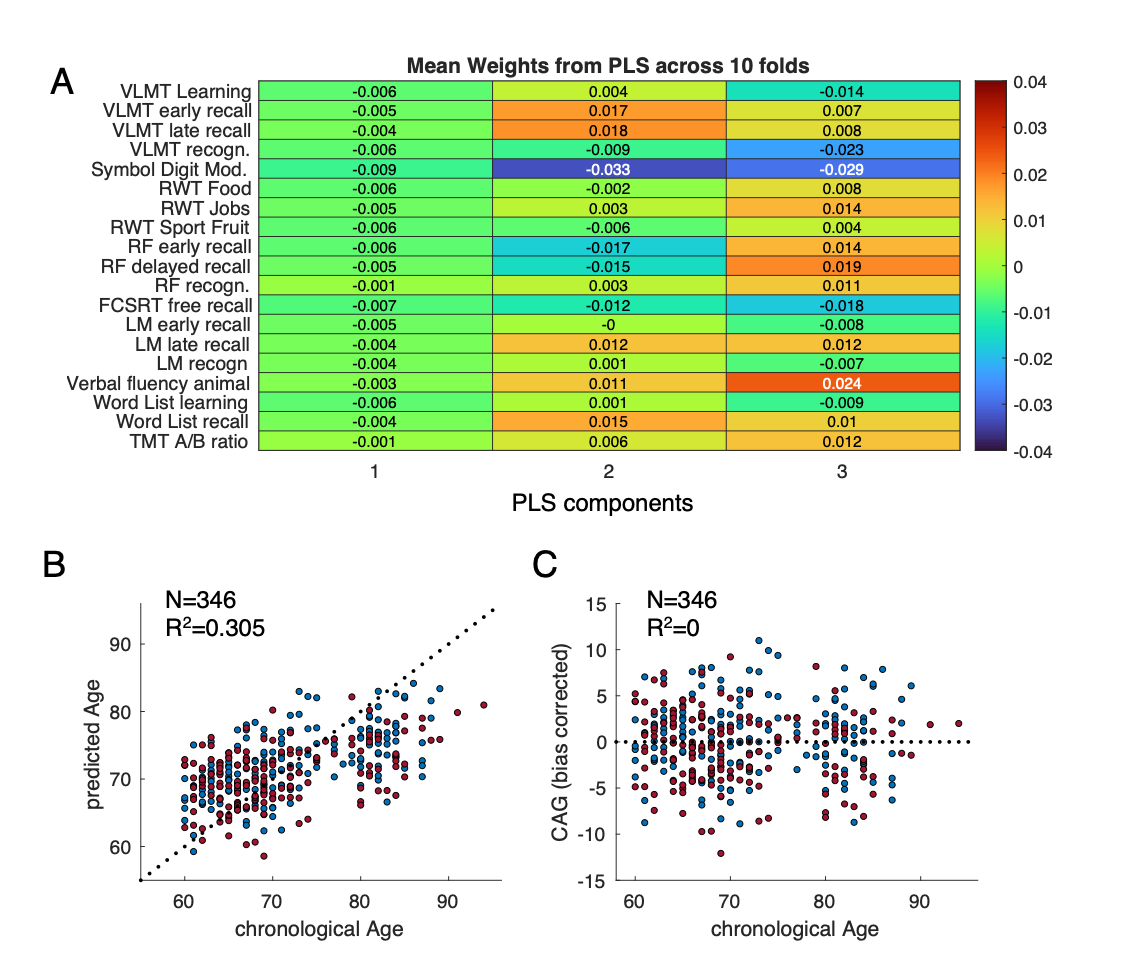


**Supplementary Fig. S5** - Results from the partial least squares model predicting cognitive age. A partial least squares regression was run to predict age by cognitive performance from 19 test scores based on the cognitive testing sessions 1 and 2. A partial least squares model with 3 components was run 10 times, leaving randomly 10% of the data out to predict age in the unseen data by model estimation in the remaining 90% of the data (10-folds). The cumulative percentage variance explained in age (average across the 10 folds) by the 19 cognitive predictor variables was 36% (*SD*=1.6) and the mean squared error (average across 10 folds) for the response variable age was 41 (*SD*=0.8. The corresponding weight (mean weight across the 10 folds) of each test score for each component is shown in (A). Component 1 was negatively weighted by all test scores (older age relates to overall worse performance), whereas component 2 showed mainly positive weightings by verbal and negative weightings by non-verbal tests. (B) Correlation plot of the association of chronological age and cognition-predicted age. A frequently observed bias in age prediction was found at the tails of the distribution, resulting in an overestimated age for younger adults and underestimated age for older adults. (C) After applying a statistical bias correction[[1]](https://paperpile.com/c/bY0ELh/vSsZS) to each individual’s predicted age, the age bias of predicted age and of the resulting cognitive age gap (CAG = chronological age - predicted age) is removed


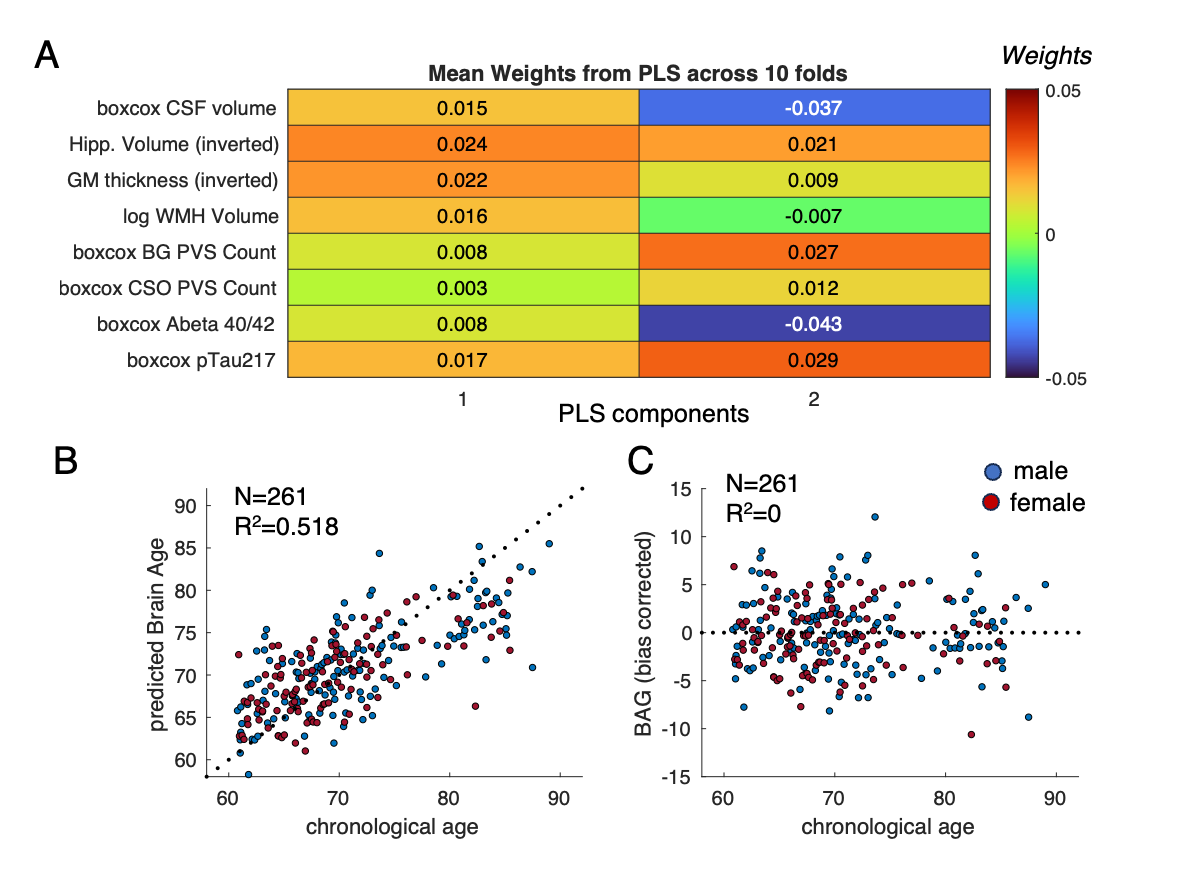


**Supplementary Fig S6** - Results from the partial least squares model predicting brain age. A partial least squares regression was run to predict age by predictors related to brain pathology/brain aging. A partial least squares model with 2 components was run 10 times, leaving randomly 10% of the data out to predict age in the unseen data by model estimation in the remaining 90% of the data (10-folds). The cumulative percentage variance explained in age by the brain predictor variables was 55% (*SD*=1.8) and the mean squared error (average across 10 folds) for the response variable age was 21 (*SD*=0.8). The corresponding weight (mean weight across the 10 folds) of each brain score for each component are shown in (A). Component 1 was positively weighted by all brain scores (older age relates to more brain pathology/degeneration). (B) Correlation plot of the association of chronological age and brain-predicted age. A frequently observed bias in age prediction was found at the tails of the distribution, resulting in an overestimated age for younger adults and underestimated age for older adults. (C) After applying a statistical bias correction[[1]](https://paperpile.com/c/bY0ELh/vSsZS) to each individual’s predicted age, the age bias of predicted age and of the resulting brain age gap (BAG = chronological age - predicted age) is removed


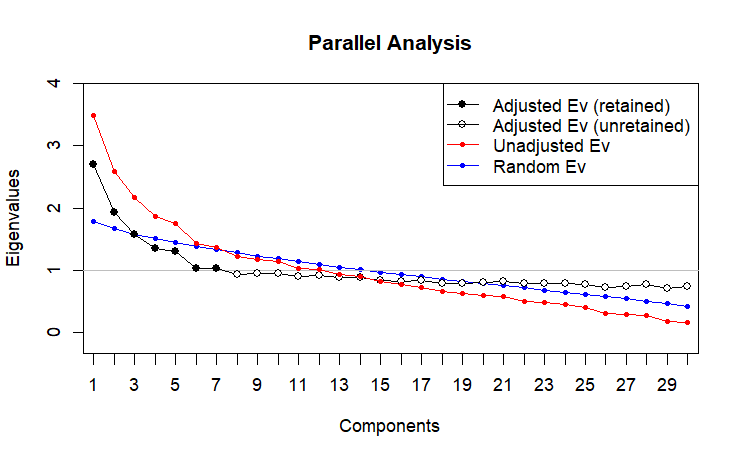


**Supplementary Fig. S7** - Horn’s parallel analysis. Horn’s parallel analysis (5000 iterations) was applied with the paran package[[2]](https://paperpile.com/c/bY0ELh/tgh9q), yielding seven components explaining more variance than expected by chance (unadjusted eigenvalue > random eigenvalue)


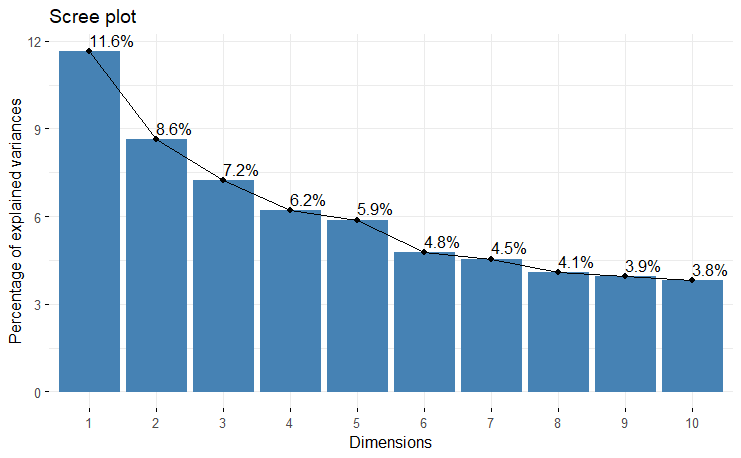


**Supplementary Fig. S8** - Scree plot of the explained variance of the first ten principal components of lifestyle and general health. The first seven components which were retained according to Horn’s parallel analysis collectively account for 48.8% of the variance. Dimensions label the principal components in the same order as discussed in the main body of the paper


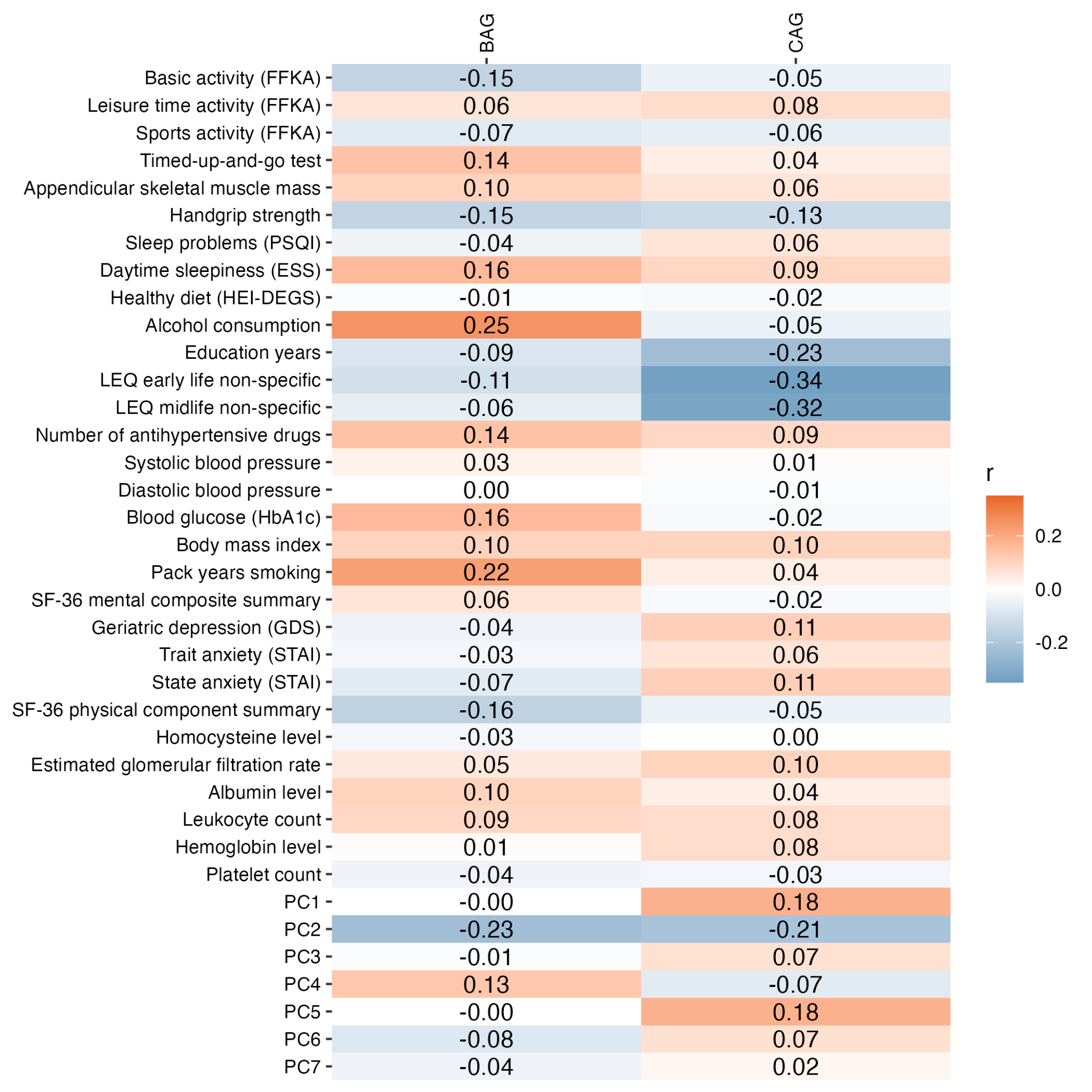


**Supplementary Fig. S9** - Pearson correlations of the lifestyle/health variables and principal components with brain age gap and cognitive age gap. Cardiovascular risk factors (alcohol consumption, pack years smoking, blood glucose, number of antihypertensive drugs, body mass index) and physical activity/fitness variables (basic activity, timed-up-and-go test, handgrip strength) show positive and negative correlations with brain age gap in a relatively consistent way, respectively. Proxies of mental stimulation and mental leisure activities throughout life (subscales of the Lifetime of Experiences Questionnaire (LEQ) and education years) are strongly associated with cognitive age gap, but also note the correlation between handgrip strength and cognitive age gap. brain age gap, BAG; cognitive age gap, CAG; principal component, PC


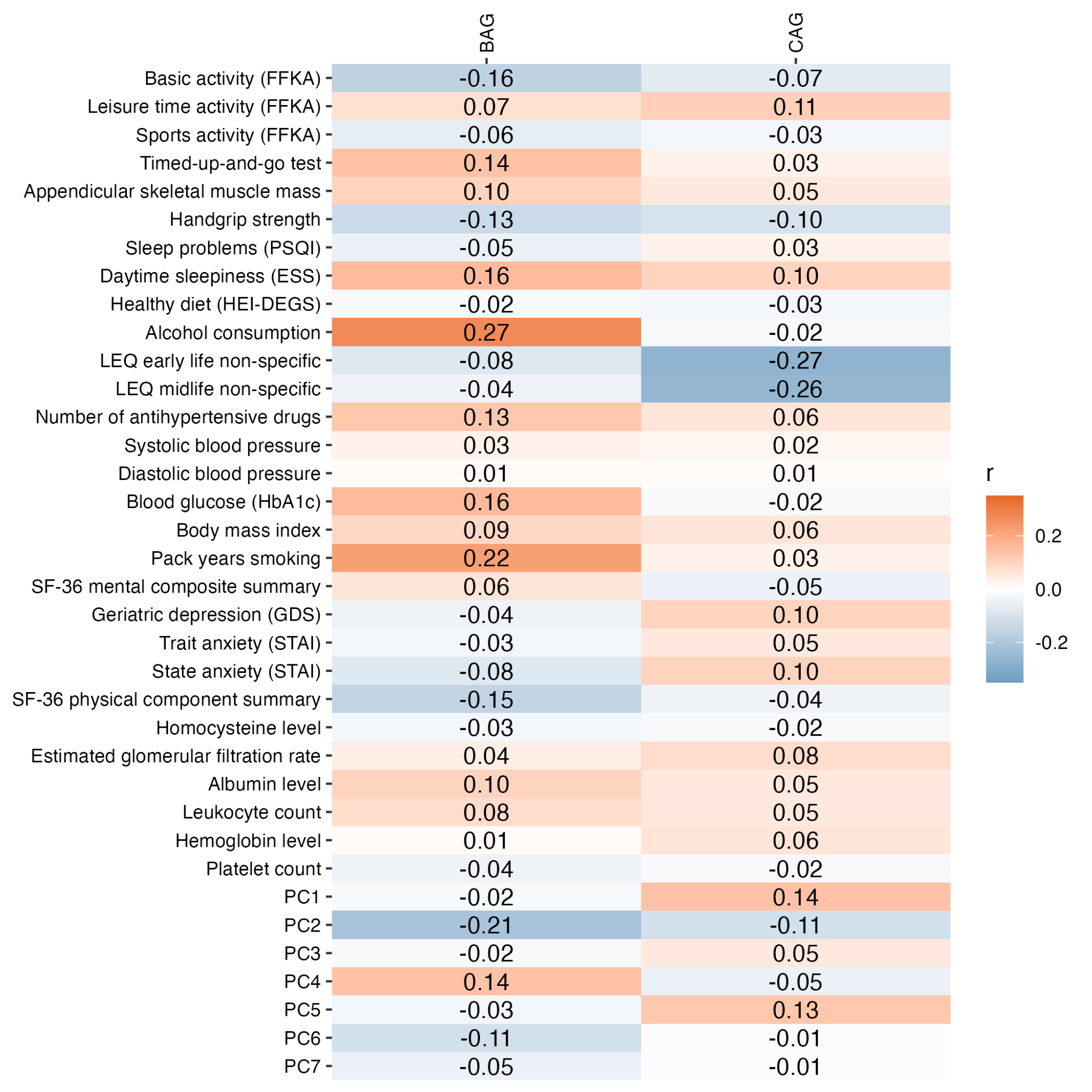


**Supplementary Fig. S10** - Pearson correlations of the lifestyle/health variables and principal components with brain age gap and cognitive age gap after regressing out education years. Compared to Supplementary Figure S8, most correlations with lifestyle/health variables only change marginally except for the subscales of the Lifetime of Experiences questionnaire (LEQ). Even though we selected the non-specific subscale, which does not contain scores based on the educational background, educational background is expected to shape the engagement in mental leisure time activities to some extent (and socioeconomic status might be a covarying factor). Consequently, it is noteworthy that the correlations of the LEQ subscales with cognitive age gap weakened but remained on a high level, suggesting an association beyond mere number of education years. brain age gap, BAG; cognitive age gap, CAG; principal component, PC

##

## Supplementary Tables

**Supplementary Table 1 - Sample characteristics of the CAG-only and CAG-and-BAG sample (referred to as BAG subsample in the main body).**

|  | **CAG-only sample**  **(*n*=35)** | **CAG-and-BAG sample**  **(*n*=171)** |
| --- | --- | --- |
| **Age (years)** |  |  |
| Mean (*SD*), [Min, Max] | 73.9 (9.0), [60.9, 88.9] | 70.2 (6.88), [60.3, 87.1] |
| **Sex** |  |  |
| Female/male (%) | 18/17 (51/49%) | 78/93 (46/54%) |
| **Education (years)** |  |  |
| Mean (*SD*), [Min, Max] | 14.3 (2.7), [10.0, 20.0] | 14.9 (2.30), [11.0, 20.0] |
| **MMSE z-value** |  |  |
| Mean (*SD*), [Min, Max] | -0.09 (0.88), [-2.51, 1.49] | 0.04 (0.87), [-2.19, 1.77] |
| **CERAD+ z-value** |  |  |
| Mean (*SD*), [Min, Max] | 0.48 (0.37), [-0.20, 1.51] | 0.48 (0.39), [-0.42, 1.61] |
| **GDS-30** |  |  |
| Mean (*SD*), [Min, Max] | 3.9 (3.3), [0.0, 13.0] | 4.0 (4.3), [0.0, 24.0] |
| ***APOE* ε4** |  |  |
| Carrier/non-carrier (%) | 8/26 (23/74%)* | 40/130 (23/76%)* |
| **Aβ_1-42_/Aβ_1-40_** |  |  |
| Mean (*SD*), [Min, Max] | 0.088 (0.012), [0.061, 0.108]* | 0.091 (0.011), [0.057, 0.113] |
| **pTau_217_ (pg/ml)** |  |  |
| Mean (*SD*), [Min, Max] | 0.157 (0.123), [0.058, 0.684]* | 0.125 (0.082), [0.047, 0.678] |

*APOE* ε4 “carrier” represents individuals with at least one ε4 allele; one subject had a z-score of -2.51 with a raw score of 25 in the MMSE, but was cleared by the Cognition Expert Panel based on otherwise normal congruent test results; cognitive age gap, CAG; brain age gap, BAG; Mini Mental State Examination, MMSE; Consortium to Establish a Registry for Alzheimer’s Disease, CERAD; Geriatric Depression Scale, GDS; Apolipoprotein E, *APOE*; Amyloid-beta, Aβ; phospho-tau, pTau; * data of 1 subject missing. Only age differed significantly between the CAG-only and CAG-and-BAG sample at a significance level of *p*≤0.01 (two-sided t-test, equal variances).

**Supplementary Table 2 - Results of the linear regressions of cognitive age gap and brain age gap on the lifestyle/health principal components with education years as covariate.**

|  | **Cognitive age gap** | | | **Brain age gap** | | |
| --- | --- | --- | --- | --- | --- | --- |
| *Predictors* | *Estimates* | *CI* | *p* | *Estimates* | *CI* | *p* |
| (Intercept) | *-0.69* | *-1.44 – 0.06* | *0.071* | *-0.75* | *-1.56 – 0.06* | *0.071* |
| PC1 | **0.32** | **0.04 – 0.61** | **0.027** | -0.03 | -0.32 – 0.27 | 0.863 |
| PC2 | **-0.56** | **-0.98 – -0.15** | **0.008** | **-0.44** | **-0.87 – -0.01** | **0.044** |
| PC3 | 0.13 | -0.23 – 0.48 | 0.489 | -0.07 | -0.44 – 0.31 | 0.721 |
| PC4 | -0.22 | -0.60 – 0.17 | 0.268 | *0.37* | *-0.02 – 0.76* | *0.063* |
| PC5 | **0.45** | **0.03 – 0.87** | **0.036** | -0.09 | -0.53 – 0.35 | 0.685 |
| PC6 | 0.09 | -0.39 – 0.58 | 0.703 | -0.30 | -0.81 – 0.22 | 0.258 |
| PC7 | 0.13 | -0.32 – 0.57 | 0.582 | -0.10 | -0.57 – 0.37 | 0.675 |
| Sex [male] | **1.57** | **0.54 – 2.60** | **0.003** | **1.13** | **0.02 – 2.24** | **0.046** |
| Education years | -0.29 | -1.08 – 0.50 | 0.469 | -0.24 | -1.08 – 0.60 | 0.579 |
|  | Observations | *n*=206 |  | Observations | *n*=171 |  |
|  | *R^2^*=0.181 |  |  | *R^2^*=0.099 |  |  |

Results of linear regression for cognitive age gap (*n*=206) and brain age gap (*n*=171) as dependent variables each. Predictors included the lifestyle/health principal components: *Low Mental Health (PC1)*, *Active Life (PC2)*, *Acute High Blood Pressure (PC3)*, *Robust Physique & High Alcohol consumption (PC4)*, *Mentally Inactive & Physically Active (PC5), PC6, and PC7, as well as sex and education.* Compared to the results reported in Table 2, the associations were weakened but remained significant. Bold font indicates significant effects (*p*<0.05); font in italics marks trends (*p*<0.1); 95%-confidence interval, *CI*; total variance explained, *R^2^*; Principal component, PC.

**Supplementary Table 3 - Sample characteristics of the combined CAG and APOE sample.**

|  | ***APOE* ε4 carrier (*n*=72)** | ***APOE* ε4 non-carrier (*n*=229)** | **Total CAG-*APOE* sample (*n*=301)** |
| --- | --- | --- | --- |
| **Age (years)** |  |  |  |
| Mean (*SD*), [Min, Max] | 71.9 (8.66), [61.1, 91.2] | 71.3 (7.56), [60.3, 89.1] | 71.4 (7.83), [60.3, 91.2] |
| **Sex** |  |  |  |
| Female/male (%) | 38/34 (53/47%) | 102/127 (46/54%) | 140/161 (47/53%) |
| **Education (years)** |  |  |  |
| Mean (*SD*), [Min, Max] | 14.9 (2.4), [11.0, 20.0] | 15.1 (2.4), [8.00, 20.0] | 15.0 (2.4), [8.00, 20.0] |
| **MMSE z-value** |  |  |  |
| Mean (*SD*), [Min, Max] | -0.08 (0.95), [-2.19, 1.51] | -0.01 (0.85), [-2.51, 1.77] | -0.03 (0.90), [-2.51, 1.77] |
| **CERAD+ z-value** |  |  |  |
| Mean (*SD*), [Min, Max] | 0.44 (0.46), [-0.610, 1.61] | 0.47 (0.39), [-0.460, 1.99] | 0.47 (0.41), [-0.610, 1.99] |
| **GDS-30** |  |  |  |
| Mean (*SD*), [Min, Max] | 3.9 (3.6), [0, 17.0] | 4.0 (4.1), [0, 24.0]* | 4.0 (4.0), [0, 24.0]* |
| **Aβ_1-42_/Aβ_1-40_** |  |  |  |
| Mean (*SD*), [Min, Max] | 0.084 (0.012), [0.0609, 0.113] | 0.089 (0.011), [0.0359, 0.122]^†^ | 0.088 (0.012), [0.0359, 0.122]^†^ |
| **pTau_217_ (pg/ml)** |  |  |  |
| Mean (*SD*), [Min, Max] | 0.175 (0.134), [0.0610, 0.692] | 0.119 (0.0738), [0.0460, 0.556]^†^ | 0.133 (0.0948), [0.0460, 0.692]^†^ |

*APOE* ε4 “carrier” represents individuals with at least one ε4 allele; cognitive age gap, CAG; Mini Mental State Examination, MMSE; Consortium to Establish a Registry for Alzheimer’s Disease, CERAD; Geriatric Depression Scale, GDS; Apolipoprotein E, *APOE*; Amyloid-beta, Aβ; phospho-tau, pTau; * data of 2 subjects missing; ^†^ data of 1 subject missing. Only Aβ_1-42_/Aβ_1-40_ and pTau_217_ differed significantly between *APOE* ε4 carriers and non-carriers at a significance level of *p*≤0.001 (two-sided t-test, equal variances).

**Supplementary Table 4 - Sample characteristics of the combined BAG and *APOE* sample.**

|  | ***APOE* ε4 carrier (*n*=58)** | ***APOE* ε4 non-carrier (*n*=177)** | **Total BAG-*APOE* sample (*n*=235)** |
| --- | --- | --- | --- |
| **Age (years)** |  |  |  |
| Mean (*SD*), [Min, Max] | 70.2 (6.9), [61.1, 88.9] | 70.3 (6.9), [60.3, 87.4] | 70.3 (6.9), [60.3, 88.9] |
| **Sex** |  |  |  |
| Female/male (%) | 31/27 (53/47%) | 76/101 (43/57%) | 107/128 (46/54%) |
| **Education (years)** |  |  |  |
| Mean (*SD*), [Min, Max] | 15.2 (2.4), [12.0, 20.0] | 15.2 (2.3), [10.0, 20.0] | 15.2 (2.3), [10.0, 20.0] |
| **MMSE z-value** |  |  |  |
| Mean (*SD*), [Min, Max] | -0.13 (1.00), [-2.2, 1.5] | 0.00 (0.84), [-1.7, 1.8] | -0.03 (0.88), [-2.2, 1.8] |
| **CERAD+ z-value** |  |  |  |
| Mean (*SD*), [Min, Max] | 0.48 (0.47), [-0.39, 1.6] | 0.47 (0.37), [-0.46, 1.4] | 0.47 (0.39), [-0.46, 1.6] |
| **GDS-30** |  |  |  |
| Mean (*SD*), [Min, Max] | 3.3 (3.0), [0, 12.0] | 3.9 (4.3), [0, 24.0] | 3.8 (4.0), [0, 24.0] |
| **Aβ_1-42_/Aβ_1-40_** |  |  |  |
| Mean (*SD*), [Min, Max] | 0.084 (0.013), [0.044, 0.113] | 0.090 (0.011), [0.036, 0.110] | 0.088 (0.012), [0.036, 0.113] |
| **pTau_217_ (pg/ml)** |  |  |  |
| Mean (*SD*), [Min, Max] | 0.157 (0.107), [0.061, 0.678] | 0.118 (0.077), [0.046, 0.556] | 0.128 (0.087), [0.046, 0.678] |

*APOE* ε4 “carrier” represents individuals with at least one ε4 allele; cognitive age gap, CAG; Mini Mental State Examination, MMSE; Consortium to Establish a Registry for Alzheimer’s Disease, CERAD; Geriatric Depression Scale, GDS; Apolipoprotein E, *APOE*; Amyloid-beta, Aβ; phospho-tau, pTau; * data of 2 subjects missing; ^†^ data of 1 subject missing. Only Aβ_1-42_/Aβ_1-40_ and pTau_217_ differed significantly between *APOE* ε4 carriers and non-carriers at a significance level of *p*≤0.01 (two-sided t-test, equal variances).

## Supplementary Methods

### Visit 1 & 2 -Interview & cognitive testing

During visit 1, participants underwent the CERAD-Plus (Consortium to Establish a Registry for Alzheimer’s Disease[[3]](https://paperpile.com/c/bY0ELh/LSgMR)) Neuropsychological Assessment Battery. In addition to the exclusion criterion of a MMSE score below 26, the following more comprehensive exclusion criteria were defined by the multidisciplinary study team: a z-score smaller than -1.5 *SD* in at least one of the tests from the CERAD-Plus test battery and evidence of possible cognitive limitations based on the neuropsychological Jak/Bondi criteria. An exclusion due to Jak/Bondi criteria was warranted if participants exhibited either (i) at least two test performances below -1 *SD* within the same cognitive domain or (ii) at least one test performance below -1 *SD* in at least one test across each of the three cognitive domains:

Memory: Word List Total; Word List Delayed Recall; Figures - Savings

Language: Boston Naming Test (short form); Verbal Fluency (animals); Verbal Fluency (S-words)

Visual-motor skills: Trail Making Test A (TMT-A); Trail Making Test B (TMT-B); Figures - Copy

During visit 2, further comprehensive cognitive testing across different cognitive domains was conducted: Verbal Learning and Memory Test (VLMT)[[4]](https://paperpile.com/c/bY0ELh/8icTc), Logical Memory II of the Wechsler Memory Scale (WMS)[[5]](https://paperpile.com/c/bY0ELh/d8yEU), German picture version of the Free and Cued Selective Reminding Test (FCSRT)[[6]](https://paperpile.com/c/bY0ELh/kDFjB), Rey Complex Figure Test and Recognition Trial (RCFT)[[7]](https://paperpile.com/c/bY0ELh/j1S5E), Symbol Digit Modalities Test (SDMT)[[8]](https://paperpile.com/c/bY0ELh/XCMN8), Go/No-go task of the Test battery for Attentional Performance[[9]](https://paperpile.com/c/bY0ELh/EAzfL), and German Regensburger Wortflüssigkeitstest (RWT)[[10]](https://paperpile.com/c/bY0ELh/BcBUX).

### MRI and PET scanning

The following absolute/relative contraindications applied to the MRI and PET measurements:

Absolute contraindications for MRI: cardiac implantable electronic device (e.g., pacemaker, cardiac defibrillators), implantable neurostimulation systems, cerebral artery aneurysm clips, cochlear implants, drug infusion pump, metal coils/stents, metallic fragments (e.g., metal shrapnel), magnetic dental implants, claustrophobia, acute tinnitus; relative contraindications for MRI (eligibility assessed by MRI team): tattoos, permanent make-up, passive medical implants (e.g., hip/knee implants).

Absolute contraindications for PET: severe liver disease, kidney disease requiring dialysis, history of seizures (e.g. epilepsy), major illness, intake of psychiatric medication, drug abuse, conditions that make it difficult to lie still during imaging, fever; relative contraindications PET (eligibility assessed by PET team): radiation exposure within the last 10 years (e.g., radiotherapy), insulin required diabetes mellitus, hyper-/hypothyroidism, allergies, intake of certain medication.

### MRI processing

T1-weighted MPRAGE scans were segmented using FreeSurfer version 7.1[[11]](https://paperpile.com/c/bY0ELh/odLkK) using the Desikan–Killiany atlas[[12]](https://paperpile.com/c/bY0ELh/a4KXE). TICV was estimated using FreeSurfer’s SAMSEG-based structural segmentation[[13]](https://paperpile.com/c/bY0ELh/F61GM). WMH were segmented using T1-weighted and FLAIR images and the AI-enhanced version of the Lesion Segmentation Toolbox (LST-AI)[[14–16]](https://paperpile.com/c/bY0ELh/AQN86+GCdPU+wz4xQ) and the USCLobes Atlas[[17]](https://paperpile.com/c/bY0ELh/CgkFc).

### Lifetime of Experience Questionnaire

The Lifetime of Experience Questionnaire (LEQ) is divided into three scales covering the age ranges 13-30 years (early life), 30-65 years (midlife), and 65+ years (late life). All scales contain a specific and a non-specific scale. The non-specific scale consists of six 6-point Likert scale items and one multiple-choice item (8 options) asking about the frequency with which the respondent saw family and friends, played an instrument, was artistically active, engaged in physical activity, spent time reading, practiced a second language, and the number of continents that have been traveled in the respective age range. The specific scale partially differed between the age ranges, containing school and post-secondary degrees in early life, post-secondary degrees, occupation and managerial capacity in midlife, post-secondary degrees and a set of questions focused on daily (mental) leisure activities in late life. Because many of our participants were under 65 and unable to complete the late life scale, we did not include it in our analyses. In addition, we found that many participants had difficulty answering the questions about their post-secondary degrees, occupation, and managerial capacity, which may have been due to a combination of reasons, including problems understanding the layout, problems understanding the classification scheme of occupational groups, lack of clarity in the definition of managerial capacity, and a lack of adaptation of the response categories of post-secondary degrees to East German educational pathways. In addition, since educational differences are already captured by years of education, which we independently included in our pool of lifestyle variables, we only included the non-specific scales of early life and midlife scales from the LEQ.

### Statistical analyses

The univariate normality of our variables was assessed visually and with the MVN package[[18]](https://paperpile.com/c/bY0ELh/k7jn1). Variables that were nor normally distributed were transformed with the functions of the bestNormalize package[[19]](https://paperpile.com/c/bY0ELh/NDyZM). Extreme outliers were identified with the identify_outliers function of the rstatix package[[20]](https://paperpile.com/c/bY0ELh/i1p4X). Missing data was imputed with the missForest package[[21]](https://paperpile.com/c/bY0ELh/BqlhN). Horn’s parallel analysis (5000 iterations) was conducted using the paran package[[2]](https://paperpile.com/c/bY0ELh/tgh9q). Causal mediation analysis was conducted using the mediation package[[22]](https://paperpile.com/c/bY0ELh/qCAbV).

# References:

[1. Beheshti I, Nugent S, Potvin O, Duchesne S. Bias-adjustment in neuroimaging-based brain age frameworks: A robust scheme. Neuroimage Clin [Internet]. 2019;24:102063. Available from:](http://paperpile.com/b/bY0ELh/vSsZS) <http://dx.doi.org/10.1016/j.nicl.2019.102063>

[2. Dinno A. paran: Horn’s Test of Principal Components/Factors [Internet]. CRAN: Contributed Packages. The R Foundation; 2007. Available from:](http://paperpile.com/b/bY0ELh/tgh9q) <http://dx.doi.org/10.32614/cran.package.paran>

[3. Schmid NS, Ehrensperger MM, Berres M, Beck IR, Monsch AU. The Extension of the German CERAD Neuropsychological Assessment Battery with Tests Assessing Subcortical, Executive and Frontal Functions Improves Accuracy in Dementia Diagnosis. Dement Geriatr Cogn Dis Extra [Internet]. 2014;4:322–34. Available from:](http://paperpile.com/b/bY0ELh/LSgMR) <http://dx.doi.org/10.1159/000357774>

[4. Helmstaedter C, Durwen HF. [The Verbal Learning and Retention Test. A useful and differentiated tool in evaluating verbal memory performance]. Schweiz Arch Neurol Psychiatr (1985) [Internet]. 1990;141:21–30. Available from:](http://paperpile.com/b/bY0ELh/8icTc) <https://www.ncbi.nlm.nih.gov/pubmed/1690447>

[5. Wechsler D. WMS-R: Wechsler Memory Scale-Revised : Manual [Internet]. Psychological Corporation; 1987. Available from:](http://paperpile.com/b/bY0ELh/d8yEU) <https://books.google.com/books/about/WMS_R.html?hl=&id=Q2RIPwAACAAJ>

[6. Buschke H. Cued recall in amnesia. J Clin Neuropsychol [Internet]. 1984;6:433–40. Available from:](http://paperpile.com/b/bY0ELh/kDFjB) <http://dx.doi.org/10.1080/01688638408401233>

[7. Meyers JE, Meyers KR. Rey complex figure test under four different administration procedures. Clin Neuropsychol [Internet]. 1995;9:63–7. Available from:](http://paperpile.com/b/bY0ELh/j1S5E) <http://www.tandfonline.com/doi/abs/10.1080/13854049508402059>

[8. Smith A. Symbol Digit Modalities Test [Internet]. PsycTESTS Dataset. American Psychological Association (APA); 2016. Available from:](http://paperpile.com/b/bY0ELh/XCMN8) <https://doi.apa.org/doi/10.1037/t27513-000>

[9. Zimmermann P, Fimm B. Testbatterie zur Aufmerksamkeitsprüfung : TAP, Version 2.2. Psytest, Herzogenrath; 2009.](http://paperpile.com/b/bY0ELh/EAzfL)

[10. Aschenbrenner S, Tucha O, Lange KW. Regensburger wortflüssigkeits-test: RWT. Hogrefe, Verlag für Psychologie; 2000.](http://paperpile.com/b/bY0ELh/BcBUX)

[11. Fischl B. FreeSurfer. Neuroimage [Internet]. 2012;62:774–81. Available from:](http://paperpile.com/b/bY0ELh/odLkK) <http://dx.doi.org/10.1016/j.neuroimage.2012.01.021>

[12. Fischl B, van der Kouwe A, Destrieux C, Halgren E, Ségonne F, Salat DH, et al. Automatically parcellating the human cerebral cortex. Cereb Cortex [Internet]. 2004;14:11–22. Available from:](http://paperpile.com/b/bY0ELh/a4KXE) <http://dx.doi.org/10.1093/cercor/bhg087>

[13. Puonti O, Iglesias JE, Van Leemput K. Fast and sequence-adaptive whole-brain segmentation using parametric Bayesian modeling. Neuroimage [Internet]. 2016;143:235–49. Available from:](http://paperpile.com/b/bY0ELh/F61GM) <http://dx.doi.org/10.1016/j.neuroimage.2016.09.011>

[14. Isensee F, Schell M, Pflueger I, Brugnara G, Bonekamp D, Neuberger U, et al. Automated brain extraction of multisequence MRI using artificial neural networks. Hum Brain Mapp [Internet]. 2019;40:4952–64. Available from:](http://paperpile.com/b/bY0ELh/AQN86) <http://dx.doi.org/10.1002/hbm.24750>

[15. Wiltgen T, McGinnis J, Schlaeger S, Kofler F, Voon C, Berthele A, et al. LST-AI: a Deep Learning Ensemble for Accurate MS Lesion Segmentation [Internet]. medRxiv. 2024. Available from:](http://paperpile.com/b/bY0ELh/GCdPU) <http://dx.doi.org/10.1101/2023.11.23.23298966>

[16. Yushkevich PA, Pluta J, Wang H, Wisse LEM, Das S, Wolk D. IC‐P‐174: Fast automatic segmentation of hippocampal subfields and medial temporal lobe subregions in 3 Tesla and 7 Tesla T2‐weighted MRI. Alzheimers Dement [Internet]. 2016;12:P126–7. Available from:](http://paperpile.com/b/bY0ELh/wz4xQ) <https://alz-journals.onlinelibrary.wiley.com/doi/10.1016/j.jalz.2016.06.205>

[17. Joshi AA, Choi S, Liu Y, Chong M, Sonkar G, Gonzalez-Martinez J, et al. A hybrid high-resolution anatomical MRI atlas with sub-parcellation of cortical gyri using resting fMRI. J Neurosci Methods [Internet]. 2022;374:109566. Available from:](http://paperpile.com/b/bY0ELh/CgkFc) <http://dx.doi.org/10.1016/j.jneumeth.2022.109566>

[18. Korkmaz S, Goksuluk D, Zararsiz G. MVN: An R package for assessing multivariate normality. R J [Internet]. 2014;6:151. Available from:](http://paperpile.com/b/bY0ELh/k7jn1) <http://dx.doi.org/10.32614/rj-2014-031>

[19. Peterson R. Finding optimal normalizing transformations via bestNormalize. R J [Internet]. 2021;13:310. Available from:](http://paperpile.com/b/bY0ELh/NDyZM) <http://dx.doi.org/10.32614/rj-2021-041>

[20. Kassambara A. rstatix: Pipe-Friendly Framework for Basic Statistical Tests [Internet]. CRAN: Contributed Packages. The R Foundation; 2019. Available from:](http://paperpile.com/b/bY0ELh/i1p4X) <http://dx.doi.org/10.32614/cran.package.rstatix>

[21. Stekhoven DJ. MissForest: Nonparametric missing value imputation using random forest [Internet]. CRAN: Contributed Packages. The R Foundation; 2011. Available from:](http://paperpile.com/b/bY0ELh/BqlhN) <http://dx.doi.org/10.32614/cran.package.missforest>

[22. Tingley D, Yamamoto T, Hirose K, Keele L, Imai K, Trinh M, et al. Mediation: Causal mediation analysis [Internet]. CRAN: Contributed Packages. The R Foundation; 2009. Available from:](http://paperpile.com/b/bY0ELh/qCAbV) <https://cran.r-project.org/package=mediation>
